# Auditory evoked potentials: Differences by sex, race, and menstrual cycle and correlations with common psychoacoustical tasks

**DOI:** 10.1371/journal.pone.0251363

**Published:** 2021-05-12

**Authors:** Dennis McFadden, Craig A. Champlin, Michelle H. Pho, Edward G. Pasanen, Mindy M. Maloney, Erin M. Leshikar

**Affiliations:** 1 Department of Psychology, Center for Perceptual Systems, University of Texas, Austin, Texas, United States of America; 2 Department of Speech, Language, and Hearing Sciences, University of Texas, Austin, Texas, United States of America; University of Leeds, UNITED KINGDOM

## Abstract

Auditory brainstem responses (ABRs) and auditory middle-latency responses (AMLRs) to a click stimulus were measured in about 100 subjects. Of interest were the sex differences in those auditory evoked potentials (AEPs), the correlations between the various AEP measures, and the correlations between the AEP measures and measures of otoacoustic emissions (OAEs) and behavioral performance also measured on the same subjects. Also of interest was how the menstrual cycle affected the various AEP measures. Most ABR measures and several AMLR measures exhibited sex differences, and many of the former were substantial. The sex differences tended to be larger for latency than for amplitude of the waves, and they tended to be larger for a weak click stimulus than for a strong click. The largest sex difference was for Wave-V latency (effect size ~1.2). When subjects were dichotomized into Non-Whites and Whites, the race differences in AEPs were small within sex. However, sex and race interacted so that the sex differences often were larger for the White subjects than for the Non-White subjects, particularly for the latency measures. Contrary to the literature, no AEP measures differed markedly across the menstrual cycle. Correlations between various AEP measures, and between AEP and OAE measures, were small and showed no consistent patterns across sex or race categories. Performance on seven common psychoacoustical tasks was only weakly correlated with individual AEP measures (just as was true for the OAEs also measured on these subjects). AMLR Wave Pa unexpectedly did not show the decrease in latency and increase in amplitude typically observed for AEPs when click level was varied from 40 to 70 dB nHL (normal Hearing Level). For the majority of the measures, the variability of the distribution of scores was greater for the males than for the females.

## I. Introduction

This is the third and final report on a large-scale study designed with several goals. Multiple crews of young-adult listeners were tested behaviorally and physiologically for several weeks each. More than three years were required to test all the crews. In [1], we reported on sex, race, and menstrual-cycle differences measured on seven common psychoacoustical tasks. In [2], we reported on sex, race, and menstrual-cycle differences in various types of otoacoustic emissions (OAEs) and on the correlations between those OAEs and performance on the seven psychoacoustical tasks discussed in the first paper. In general, those correlations were small; that is, the individual differences in OAEs did not co-vary strongly with the individual differences in psychoacoustical performance [2].

For this third report, the focus is on the two auditory evoked potentials (AEPs) known as the auditory brainstem response (ABR) and the auditory middle-latency response (AMLR). ABRs and AMLRs are electrical responses from subcortical auditory brain regions, recorded using a set of electrodes attached to the scalp and earlobes. After the presentation of an acoustic click of moderate strength, a series of peaks or waves can be detected using such electrodes. The AEP peaks are traditionally categorized according to their latency following the click; peaks from 0–15 ms are called ABRs, peaks from 15–100 ms are called AMLRs, and peaks longer than about 100 ms are called auditory late responses (ALRs) (e.g., see [3]). The auditory literature now contains several measures called AEPs, but here that term will be used to denote only the click-evoked ABRs and AMLRs described here.

### A. Experimental objectives

The experimental objectives of the overall study were established primarily for the OAE and behavioral measures and then carried over to this AEP segment of the study. There was a hierarchy of questions. At the top were the questions of whether OAEs covaried with psychoacoustical performance and how sex differences and menstrual-cycle differences affected those covariations. Also of interest was whether OAE strength correlated with AEP strength (did any relationships seen for OAEs propagate from the cochlea into neural activity?). Because AEPs were to be measured for the latter purpose, it was logical to examine the AEP responses in the same framework as used for the OAE and behavioral measures; namely, to examine the covariation between the various AEP and psychoacoustical measures and to document how sex differences and menstrual-cycle differences affected those covariations. Twenty-two measures of latency and amplitude were obtained, 8 for ABR and 14 for AMLR (detailed below). Having those various AEP measures also allowed an examination of their interrelationships. During the analysis of the behavioral and physiological data, race/ethnicity emerged as a relevant variable, and all the planned analyses were expanded to include comparisons by race [1, 2].

Concretely then, for AEPs the experimental goals were to:

#### (1) Determine the existence and magnitude of the sex differences in latency and amplitude of the various peaks in the ABR and AMLR responses

Auditory science long has been aware of sex differences in certain AEPs. For ABRs, the typical findings are that the latency of Wave V is shorter in females than males, and the amplitudes of Waves I and V are more robust in females than males (e.g., [3–7]). There is evidence that these sex differences in ABRs are not solely attributable to the generally smaller head size in females [8–11, but compare 3, 12] and instead may be attributable to sex differences in the cochlea [9] and subcortical neural differences [11].

Existing evidence suggests that the sex difference in Wave V does not exist in newborns but does exist prior to puberty [3, 11]. To the extent that cochlear differences contribute to the sex differences in AEPs, the implication is that something develops differently in the base of the cochlea for one or both sexes because the peaks of the ABR commonly are attributed to neural activity originating from the basal (high-frequency) half of the basilar membrane [13]. To the extent that neural differences contribute to the sex differences in AEPs, the implication is that something synaptic or axonal develops differently in the two sexes, perhaps under hormonal influence [11].

For AMLRs, past results have disagreed about the existence of sex differences. Tucker et al. [14] observed that females had shorter latencies and larger amplitudes than males for Wave Pa, but found no sex difference for either latency or amplitude for Wave Pb (not measured here). Other studies have reported sex differences for both Waves Pa and Pb; a summary exists in [14], who suggested that some of these contradictions may be attributable to the specific stimulus values used in the various studies. McFadden and Champlin [6] reported no significant sex differences for 6 AMLR measures, but 2 of 7 ALR measures did differ significantly between the sexes.

#### (2) Determine the existence and magnitude of differences across the menstrual cycle in naturally-cycling women

A substantial literature exists on the effects of the menstrual cycle on the auditory system, for behavioral measures (e.g., [15]), for AEPs (e.g., [16–19]), and for OAEs (e.g., [20, 21]). A generalization of the various results was that the ABRs of females were more different from males during the midluteal phase than during menses; see [22, 23]. The most studied AEP measure was Wave V of the ABR, and we planned to verify those outcomes while asking if any peaks of the AMLR (higher brain locations) also varied with the cycle.

Note that the basic sex differences in AEPs are not attributable to the menstrual cycle; they exist in life prior to the onset of menstruation and appear to be relatively constant through life. Any menstrual effects are modulations on this basic, permanent sex difference. Just as for OAEs (review in [24]), the basic sex difference in AEPs is presumed to be a permanent aftereffect of sex-specific exposure to certain hormones early in development and does not depend upon current circulating levels of hormones. Any menstrual effects presumably would depend upon currently circulating levels of certain hormones.

#### (3) Examine the relationships between the various AEP measures and the measures of behavioral performance obtained

Immediately upon their discovery [25], ABRs were recognized as having the potential to serve as objective measures of hearing sensitivity (summarized by [3]), and exploration continues for the optimal procedures for that purpose (e.g., [26]). Because this study involved measuring performance on seven psychoacoustical tasks to see if any task covaried with any OAE measure, we could ask the parallel question of the possible relationship between psychoacoustical task and the AEP measures. That is, in addition to absolute sensitivity, were any other behavioral tasks related to AEPs? Most of the psychoacoustical tasks studied here involved simultaneous or temporal masking, and, as explained in [1], most were chosen because of past evidence of their relationship to the cochlear-amplification process believed to depend upon the outer hair cells [27]. [Seeing the weak correlations between OAEs and psychoacoustical performance [2] was a surprise.]

When planning this study, the expectation was that systematic relationships between OAEs and psychoacoustical performance were more likely to be found than were systematic relationships between AEPs and behavior. The reason was that OAEs are more intimately tied to the cochlear-amplifier system than are brain potentials, and many psychoacoustical phenomena have characteristics that follow cochlear characteristics. Also, the stimuli used to evoke OAEs were more similar to the stimuli used for our psychoacoustical tasks (e.g., [28]) than were the simple click trains used to evoke AEPs. However, until the comparisons between AEPs and psychoacoustical task were made, that expectation was untested; hence this phase of the overall study. If strong relationships were found, they could be pursued further with more similar stimuli used for the behavioral and AEP measures. By comparison, the stimuli and procedures for collecting AEPs are very similar to those used to collect click-evoked OAEs.

#### (4) Report the effects of race/ethnicity on the differences by sex and menstrual cycle on AEPs

After all the behavioral and physiological data were collected, it became evident that race differences interacted so markedly with sex differences for 6 of the 7 psychoacoustical tasks and for OAEs that we were obliged to analyze those data within race as well as pooled over race [1, 2]. For consistency with those reports, we continue that analysis strategy here even though the race differences for AEPs generally were smaller than for OAEs and the psychoacoustical tasks. Ignoring the race effects would be a violation of the open-science movement [29, 30]. Because the study was not designed with race in mind, the outcomes obtained must be interpreted only as suggestive until truly experimental tests are implemented.

The effects of race are well-documented for OAEs, hearing sensitivity, and noise-induced hearing loss [1, 10, 28, 31–34]. We know of only two AEP studies showing race differences. Chan et al. [5] collected ABR data for Asian and White subjects (specifically, "Hong Kong Chinese" and "Australians," respectively). They reported sex differences within both groups but did not compare the races within sex. Using the data provided, we calculated effect sizes for race difference within-sex. The effect sizes were quite large, ranging between 1.2 and 2.8 for both sexes and both Waves I and V (the Asian subjects having shorter latencies). Second, Zakaria et al. [35] measured ABR responses to speech stimuli in males who were Malay or Chinese. There were no differences between those groups, but when those data were pooled and compared with similar data collected previously from White subjects, many of the effect sizes (our calculations) were quite large. Again, the Asian subjects had shorter latencies than the White subjects, and they also had generally larger amplitudes. A recent comprehensive handbook on AEPs mentioned the topic of race only once, as a potential non-pathological subject variable involved in otosclerosis [3].

#### (5) Examine the relationships between the various AEP measures and several measures of OAE

OAEs are measures from the cochlea and AEPs are measures from auditory neural pathways. A reasonable question is whether OAE strength is correlated with strength of any measure of ABR or AMLR. That is, is the strength of the cochlear-amplifier system [27] reflected in the neural response? To our knowledge, this question previously had not been asked. Expectations were that the ABR was more likely to be related to OAEs than was the AMLR because later responses of the AEP are "farther" from the cochlea and they involve summation across multiple brain regions [3].

#### (6) Examine the interrelationships between the various measures for ABR and AMLR

The question was whether the individual differences in any of the ABR or AMLR measures covaried with the individual differences in any of the other ABR or AMLR measures. Knowing which measures are highly correlated is potentially informative about underlying processes.

### B. Comment

Many of the various AEP comparisons made across sex, race, and menstrual cycle failed to achieve statistical significance, even some that had been significant in previously published articles. The modern open-science initiative [29, 30] argues that the reproducibility of individual scientific outcomes only can be determined if failed attempts to replicate are routinely reported along with successful replications. This approach is the only way to establish a scientific literature that is an accurate representation of reality. Because journal space is limited, a compromise between publication length and the full availability of results needs to exist. The solution adopted here is to include in this article a relatively full description of the motivation, methods, and discussion of the AEP study, but include in the Results section only some of the many outcomes and comparisons. The remaining results are available as additional text, figures, and tables in [36]. The individual data on which the summarized results are based are publicly available (see [37]).

## II. Methods

The emphasis here is on the methods used to collect and analyze the ABR and AMLR data. Only highlights of the procedures used for the psychoacoustical and OAE measurements are included here; additional details are available in [1, 2].

All aspects of this study were approved in advance by the Institutional Review Board of The University of Texas, Austin.

### A. Subjects

Subjects were primarily college students, who were recruited on campus using standard procedures. They were paid for their participation. The average age was 21 years for both females and males, and both race groups. Prior to being hired, subjects were screened for normal hearing and normal tympanometry using standard audiometric devices and procedures [1]. Because of our interest in the possible effects of the menstrual cycle on our measures, only females not using oral contraceptives or other forms of hormonal contraception were hired (called naturally-cycling females). All subjects provided written informed consent and completed an extensive questionnaire about previous noise exposure, drug use, and other topics.

Because race was not an anticipated variable in this study, the only questionnaire items about race/ethnicity were the two required by our granting agency (see section IIsupp.A in S1 File [36]). Those items, and our Ns, only allowed partitioning of our subjects into two broad categories for data analysis: Non-White and White. We acknowledge that this partitioning is inherently unsatisfactory because of the substantial heterogeneity in the two groups, but it was the best we could do with the information available. Ignoring the race differences would have been a violation of the open-science initiative. In section IV.B.3 we argue that race categories are poor, temporary proxies for what eventually will prove to be the actual variables of interest. No attempt was made to control the race categories for confounding factors such as socioeconomic status, nutrition, environmental exposures, or similar factors.

### B. General procedures

Subjects were hired as all-female or all-male crews of 6–8 listeners each. They participated in the psychoacoustical part of the study by working daily during the same 2-hour window, 5 days/week, for several weeks. They were paid an hourly wage plus a bonus upon completion of all testing. The same team of experimenters tested all crews of subjects.

Data were collected for seven psychoacoustical tasks using adaptive forced-choice procedures; because of time limitations, only the right ear was tested. (The choice of ear was based on the greater hearing sensitivity and greater strength of OAEs in right ears; see the review in [38].) The tasks were detection of a tone in the quiet, bandwidth of the auditory filter, overshoot, forward masking, two-tone (lateral) suppression, tone-on-tone masking with a distortion product present (the Greenwood effect [28]), and detection of a tone in the presence of a 10-tone masker (profile analysis). The signal typically was a 3.0-kHz tone, so all the tasks focused on the mid-frequency region. Subjects were extensively trained on the psychoacoustical tasks, and atypical blocks of trials were culled for each subject individually prior to data analysis. Additional details of the behavioral procedures and analyses are provided in [1].

Male crews worked for 6–8 weeks. Sometime early in this period, the OAEs, ABRs, and AMLRs of each subject were measured. Those individual test sessions typically were scheduled for times outside the 2-hr daily window during which the psychoacoustical data were collected. For the males, OAEs and AEPs were measured in separate test sessions, each about 1.0–1.5 hours in duration. For the males, OAEs and AEPs were measured in both ears, but only the right-ear data were used for the analyses reported here (primarily because no psychoacoustical data were collected for the left ear). The OAEs measured were spontaneous OAEs (SOAEs), click-evoked OAEs (CEOAEs), and distortion-product OAEs (DPOAEs); the collection procedures and analyses used were detailed in [2].

#### 1. Menstrual cycle

Female crews worked for 8–10 weeks, during which they kept daily diaries about their menstrual cycles. After all the data were collected, the diary entries were used to sort each individual subject’s daily psychoacoustical data into three categories: Menses, Midluteal, and the remainder (nominally Ovulatory). The methods for establishing these categories for each subject individually are detailed in section IIsupp.B.1 in S1 File [36]. Both estrogen and progesterone levels are low during menses, estrogen levels are high near ovulation, and both estrogen and progesterone levels are high during the midluteal phase. Thus, in terms of hormone levels, females are most similar to males during menses and most different from males during the midluteal phase.

Collecting the physiological data for the females was more complicated than for the males because the goal was to obtain AEP and OAE measures during both the menses and midluteal phases of the cycle. Those details also are provided in the S1 File.

For females, AEPs and OAEs both were measured in single sessions of 2.0–2.5 hours duration. The total time was shorter than for males because AEPs and OAEs were measured only on the right side of the head for females. Because female crews worked for 8–10 weeks, typically AEPs and OAEs were measured multiple times during the menses and midluteal phases of their cycle. However, scheduling difficulties and errors in accurately predicting the midluteal phase contributed to different numbers of measurements across subjects and phases. Only those subjects who provided at least one usable AEP/OAE session during the Menses phase and at least one during the Midluteal phase were included in the analyses for menstrual effects. When usable data were available for more than one instance of a cycle phase, those data were averaged. For the majority of subjects, the AEP and OAE sessions were conducted while the rest of that subject’s crew were being measured psychoacoustically. So, for most subjects, the Menses and Midluteal measurements were obtained at about the same time of day, which also was the same time of day for the majority of that subject’s psychoacoustical measurements. However, the pragmatics of scheduling forced some AEP and OAE measurements to be obtained during different daytime windows, in the evening, or on weekends.

### C. Measuring auditory evoked potentials

The same two-channel system was used for measuring ABRs and AMLRs. Four gold-plated surface electrodes were applied to each subject’s scalp, ear lobes, and forehead using the 10–20 system [39]. The skin was scrubbed, the cups of the electrodes filled with conductive paste, the electrodes carefully positioned, and surgical tape applied to hold the electrodes in place. A cloth tape measure was used to make three measurements of the head: the circumference just above the eyes, over the top from tragus to tragus, and over the top from nasion to inion. One electrode was placed on the intersection of the two top-of-the-head measures (Cz); it was connected to a Y-adapter so the electrical activity could be directed to the non-inverting channels of two identical differential amplifiers. The second and third electrodes were attached to the left and right earlobes (A1 and A2, respectively), and they provided input to the two inverting channels. The fourth electrode was placed on the forehead (Fz) and served as the common ground for both amplifiers. Electrode impedances were monitored and maintained below 5 kΩ, and within 2 kΩ of each other.

For testing, the subject was seated in a comfortable reclining chair inside an electrically shielded, sound-treated room having low illumination (the same chair and room as used for OAE testing). An insert earphone was placed in the right external ear canal (for females) or placed in the right or left ear canal pseudorandomly (for males). Subjects were instructed to keep their eyes open, to minimize muscular movement, and to keep mentally alert and engaged (especially for the AMLR measurements). The experimenter was able to monitor wakefulness using the ongoing electroencephalic waveform, and when a problem arose, the subject was asked to walk down a long hall and back (the electrodes remained in place, and impedances were checked before testing resumed).

A custom-shielded earphone (Etymotic Research, model ER-3A) was driven with a 90-μs electrical pulse of negative polarity, producing a rarefaction acoustic pulse in the ear canal. The pulses were generated with a 16-bit D/A converter (Tucker-Davis Technologies, model DA1) using a 50-kHz sampling rate. The pulses were presented at 18.1 and 7.1 clicks per second for ABR and AMLR, respectively. (The AMLR rate was a compromise; high rates are better for the earlier AMLR peaks and low rates are better for the later AMLR peaks [14, 40]).

Two channels of data were acquired simultaneously using the dual-channel ipsilateral/contralateral array and a multi-channel physiological amplifier (Tucker-Davis Technologies, model DB4/HS4) controlled by BioSig software (Tucker-Davis Technologies) and running on a desktop computer with a Windows-based operating system. The scalp-recorded potentials were amplified initially (gain = 5x) and digitized by a battery-powered unit (HS4) located inside the shielded room just adjacent to the subject. A noise-immune fiber-optic cable carried the digitized potentials outside the sound room to the DB4 control unit where the potentials were amplified again (gain = 200,000x), band-pass filtered (rejection rate = -12 dB/octave), and then converted back to analog form. The passband for filtering was 100–3000 Hz for ABRs and 10–300 Hz for AMLRs.

Data were collected during a time period (sweep) that began prior to click presentation and extended past it. For ABR, the sweep was 24 ms in duration, and the click stimulus was presented 12 ms into the sweep. For AMLR, the sweep was 120 ms in duration, and the click was presented 60 ms into the sweep. During each sweep, the voltage waveform in each channel was digitized using a 16-bit A/D converter (Tucker-Davis Technologies, model AD1) at a sampling rate of 50 kHz (ABR) or 10 kHz (AMLR). Trial-by-trial, the responses were evaluated by the BioSig software and added to the accumulating average response only if no peak in that response exceeded 70% of the full-scale voltage (+/-10 volts for both ABR and AMLR, and the same for all subjects). A run was repeated if the artifact-rejection rate was greater than 80%; this occurred for fewer than 2% of the total runs. The AD1 and DA1 were synchronized by an external trigger unit (TG6, also by Tucker-Davis Technologies).

Although stimulus presentation, sweep averaging, artifact rejection, filtering, digitizing, and waveform storage were accomplished using the commercially available Tucker-Davis hardware and software, measurement of latency and amplitude of the various AEP peaks was accomplished on a Macintosh computer using programs written in LabVIEW^@^ (National Instruments, Austin, TX) by author EGP.

For each condition, for each subject, at least 2000 sweeps were averaged for ABRs and at least 800 for AMLRs. For the first few subjects (all males), the ABR measures were collected before the AMLR measures, but after that AMLR was measured before ABR. The reason was that AMLR is more affected by drowsiness, which was less of a problem early in the AEP session.

In pilot work using 10 listeners with normal hearing who did not participate in the current study, the detectability of the click was measured in the quiet. The click levels used here were about 40 and 70 dB above the averaged detectability measured; using those estimates, here we label the clicks as 40 and 70 dB Normal Hearing Level (nHL). When each of those clicks was displayed on an oscilloscope, and the maximum amplitude of a 1.0-kHz tone was adjusted to equal the maximum amplitude of the click, the levels necessary were approximately 75 and 105 dB SPL (*re* 20 microPascal), respectively. Thus, the clicks were approximately 75 or 105 dB peak-equivalent dB SPL. Measures were obtained with the stronger click before the weaker click.

Here all analyses are based only on the data recorded from the right side of the head with the click stimulus in the right ear (the ipsilateral channel).

### D. Measurement details for ABRs and AMLRs

Of interest were two ABR peaks (I and V) and four AMLR peaks (Po, Na, Pa, and Nb—see S20 Fig in S1 File [36]). (Na and Nb are better visualized as troughs than peaks, but they commonly also are called peaks.) The intent was to measure latency for each peak for two click levels 30 dB apart. However, Wave I at 40 dB was challenging to resolve for many subjects, so measures of latency and amplitude for Wave I at 40 dB are omitted from the analyses here. That left 22 AEP measures, 8 for ABR and 14 for AMLR. For ABRs, both the latency and amplitude were measured for Wave I and Wave V for the 70-dB click, and latency and amplitude also were measured for Wave V using the 40-dB click. Also calculated for ABRs were the amplitude ratio Wave V/Wave I and the interpeak latency between Waves I and V, both for the 70-dB click only. For AMLRs, latency was measured for Wave Po, Wave Na, Wave Pa, and Wave Nb for both the 70-dB and 40-dB clicks, and three amplitude measures also were taken for both click levels: Wave Po-Na, Wave Na-Pa, and Wave Pa-Nb. Amplitudes typically were calculated from peak to following trough; the exception was the amplitude from Na to Pa (see S20 Fig in S1 File [36]). (A note in passing: Historically, AMLR Wave Po was thought to be ABR Wave V, but the two were shown to be distinct when using filter settings like those used here [3].)

One experienced judge (author MHP) measured the latencies and amplitudes of all the relevant ABR and AMLR peaks. When a waveform appeared not to contain one of the peaks of interest, the remaining peaks still were scored, as is commonly done (e.g., [11, 41]). Post-auricular muscular responses (PAMRs) were present in AMLRs obtained from six subjects (four females, two males). After discarding those waveforms in their entirety, all AMLR measures were lost for only one female subject (70-dB click, menses only) and two male subjects. Authors MHP and CAC consulted on the scoring of unusual waveforms. The authors regard this peak-by-peak analysis of the AEP data to have the strengths of flexibility and efficiency, and to be superior to using only averaged waveforms to summarize AEPs obtained from multiple subjects (see section IIsupp.G.2 in S1 File [36]).

To assess the reliability of the judge, a pseudorandom sample of 570 waveforms was re-scored while blind to the first scorings. The test/retest correlation was greater than 0.90. Details can be found in section IIsupp.D in S1 File [36].

### E. Two complications

After several all-male crews of listeners had been tested, the true rms voltmeter (VM) used for setting the levels of the stimuli for the psychoacoustical tasks malfunctioned (VMm). As a consequence, those early male crews were tested with stronger psychoacoustical stimuli than used for the remainder of the male crews and for all of the female crews; accordingly, the *psychoacoustical* data from those early crews could not be pooled with the psychoacoustical data from the later crews. Because the malfunctioning VM was not used for the physiological measurements, *all* male subjects could be included for the AEP (and OAE) analyses reported here. For consistency with the two previous reports [1, 2], the male data sometimes are partitioned between Pre-VMm and Post-VMm. For the correlational analyses with the behavioral tasks, only the Post-VMm males were included.

A second complication was that information about race was not obtained for some of the males in the Pre-VMm crews. While that is immaterial for comparisons for which the subjects are pooled over race, it does mean that the Ns for the Non-White and White males do not sum to the N for the pooled “all males.” These complications exist out of our intent always to use all of the data possible.

### F. Statistical analyses and resampling

One emphasis in our Results section is on the sex, menstrual-cycle, and race differences measured for the 22 AEP measures. Those various pairwise comparisons are summarized using effect size, which was calculated as the difference between the means of the two groups of interest divided by the square root of the weighted mean of the variances of those two distributions. By convention, effect sizes of 0.2, 0.5, and 0.8 are considered small, medium, and large, respectively [42]. Here the numerator for calculating effect size always was female minus male, Non-White minus White, or Menses minus Midluteal (see tables).

The magnitude of any effect size depends not just on the separation between the two means (the numerator), but also the sizes of the two variances that are averaged in the denominator. As discussed in section IV.G. below, the variances for our AEP measures were generally larger for the male groups than for the female groups, but there were no systematic differences by race. That is, the differences in our effect sizes by race were not the result of race differences in variance.

Effect sizes were calculated for a large number of pairwise comparisons. These are called the *actually obtained effect sizes*. To assess which of those outcomes were most *un*likely to be due to chance, a resampling technique was used. Specifically, for each pairwise comparison, the scores for all of the subjects in the two groups of interest were pooled, a random sample of scores equal to the N of one of the two groups of interest was drawn, these were viewed as representing one group and the remaining scores as representing the second group, the effect size for this resample was calculated, the process was repeated 20,000 times, the number of times the (absolute value of) a resampled effect size exceed the *actually obtained effect size* was tallied, and the tally was divided by 20,000. The resulting proportion was taken as the *implied significance* of the pairwise difference of interest. For some sex-difference comparisons, *none* of the 20,000 resampled effect sizes exceeded the actually obtained effect size; for those instances, we assigned a *p* value of < 0.0001. Because our tallies were based on the absolute values of the resampled effect sizes, our estimates of implied significance should be viewed as conservative (“two-tailed”). We have used versions of this resampling procedure in the past [1, 2, 28, 43].

Additional questions of interest for this study involved correlations of several types: between pairs of AEP measures, between the AEP and OAE measures, and between the AEP measures and performance in each of the seven psychoacoustical tasks. For those comparisons, Pearson product-moment correlations were calculated. Because of our psychoacoustical findings [1], those correlations were calculated within sex, menstrual phase, and race of the subjects as well as across those factors. A variant of our resampling procedure was used to assess which of the obtained correlations were the most *un*likely to be due to chance; details are in [2] and section IIsupp.F in S1 File [36].

The calculations of means, standard deviations (unbiased), Ns, standard errors, and effect size initially were made using Excel^®^ (Microsoft Corp., Redmond, WA) and later were verified using a purpose-written algorithm in LabVIEW^®^. The Pearson product-moment correlations were calculated using Excel^®^ and an algorithm available in LabVIEW^®^. The resampling of both effect size and correlations was accomplished using LabVIEW^®^ programs written by author EGP. The levels of implied significance achieved by individual effect sizes and correlations are indicated in the tables using the same hierarchy of symbols for each table. Ns for the various subgroups for each dependent variable are shown in the figures.

### G. Averaged waveforms

In addition to the systematic measurements of the individual peaks described above, the data for the various subject groupings also were summarized by calculating waveforms averaged across subjects. For example, all the ABR waveforms for all the Non-White Males were averaged point-by-point in time to create an averaged waveform that then could be compared with the averaged waveforms for other groups such as the Non-White Females or the White Males. These averaged waveforms were used solely as a visual summary of the results. Averaged waveforms are problematic as summaries of AEP data (see section IIsupp.G.2 in S1 File [36]).

### H. Strategy

Because showing graphs of the data obtained for all 22 AEP measures would be inefficient, we report only a subset of those means, standard errors, and Ns, and then use tables of effect sizes and correlations to summarize the sex, race, and menstrual-cycle differences for all the measures. Graphs for the measures not shown here as well as several additional tables are provided in [36]. For simplicity, here the detailed results for ABRs will be presented first (section III.A), followed by the results for AMLR (section III.B).

## III. Results

To reiterate, only data collected from the right side of the head with the earphone in the right ear were used in the analyses reported here. Hearing sensitivity is better and OAEs are stronger in right ears than left; see the review in [38]. Comparisons of the AEPs obtained from both sides of the head have been reported in [43].

### A. ABRs: Sex, race, and menstrual-cycle differences

#### 1. Overview

Of the 8 ABR measures, 6 exhibited significantly shorter latencies and larger amplitudes for the females than for the males, outcomes that are in general accord with the literature for ABR measures (e.g., [3–7, 10, 43]). When calculated *within*-sex, the vast majority of the race differences did not achieve implied significance, and there was little consistency across the sexes or click levels. However, sex and race did interact to produce sex differences of different magnitudes for the two race groups. No ABR measure differed significantly between the Menses and Midluteal phases of the menstrual cycle, in contradiction to past findings (e.g., [16–19, see reviews 22, 23]).

#### 2. Representative ABR outcome

**Fig 1:**
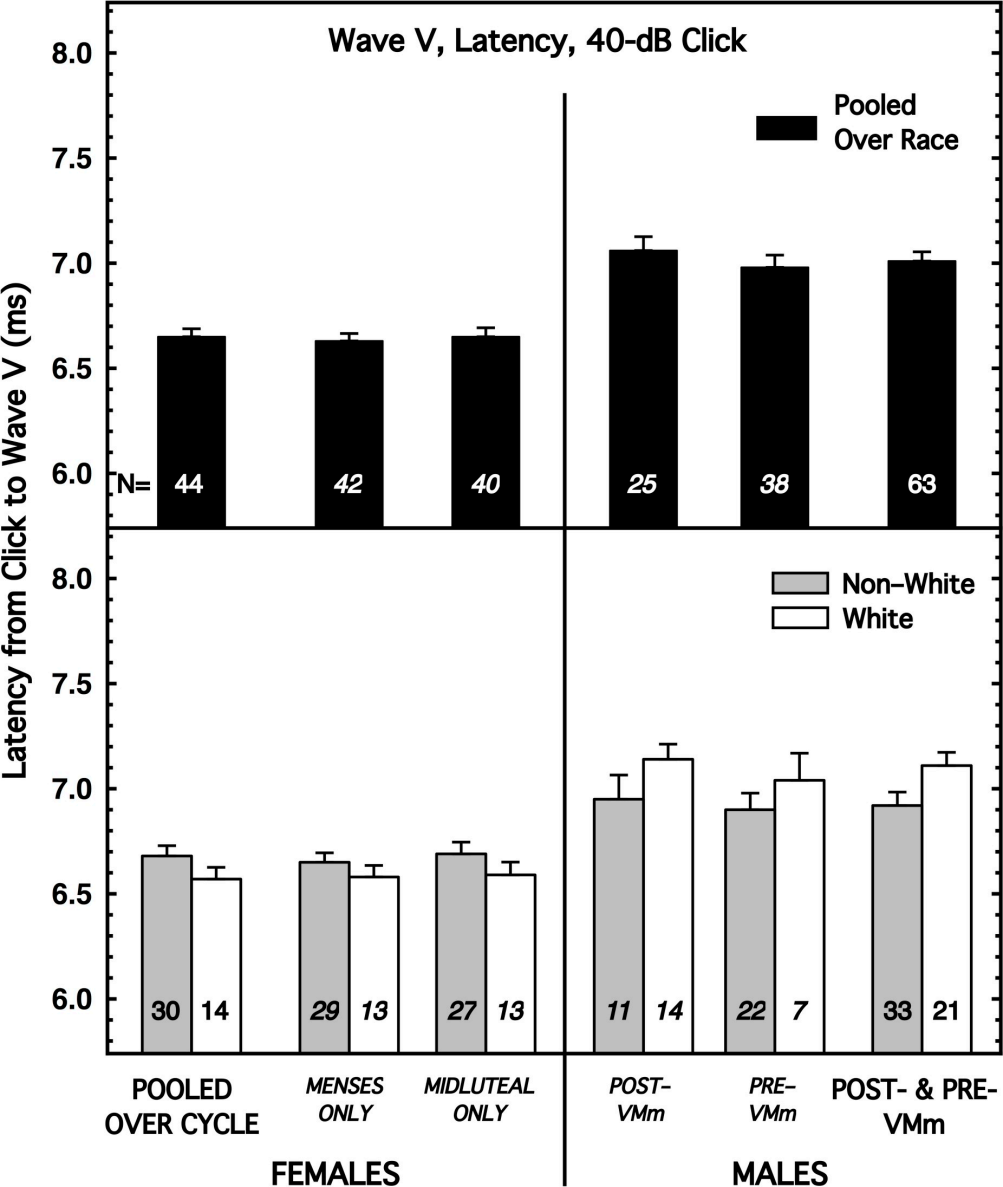
Mean latency for Wave V with the 40-dB click, shown for females on the left and males on the right. The female data are shown pooled over the menstrual cycle (far left; abscissa label not italicized) and also partitioned by cycle. The male data of most interest are at the far right (abscissa label not italicized), but, for consistency, data also are shown separately for the male subjects tested before and after the malfunction of the voltmeter (VM; which was not used for the ABR or AMLR measurements). (*top*) Subjects are pooled over race. (*bottom*) Subjects are partitioned by race. The sex difference was larger for the White subjects than for the Non-White subjects. Flags denote standard errors of the mean. Additional ABR measures are illustrated in (S1-S7 Figs in S1 File) [36].

In Fig 1, results are shown for a representative ABR measure: Wave-V latency with the 40-dB click. For consistency, the format of this figure is the same as used in [2]; this format permits easy comparison across sex, across race, across the phases of the menstrual cycle, and across the Pre-VMm and Post-VMm males. The left and right halves of the figure show results for females and males, respectively. The top and bottom halves of the figure show results for subjects pooled across race and partitioned by race, respectively. In the female panels, the *left*-most bars show the results pooled across the phases of the menstrual cycle, and the results partitioned by cycle also are shown inside those panels. In the male panels, the *right*-most bars show the results pooled across all male subjects (post-VMm plus pre-VMm males), while the results for the Post-VMm and Pre-VMm males are shown separately to the left inside those panels. Because the malfunction of the VM was not relevant for the ABR and AMLR measures, the most reliable comparisons of the two sexes are the bars at the far left of our figures *vs*. the bars at the far right, and those are the comparisons used in the primary analyses below. Similar figures are shown for each of the other ABR measures in (S1–S7 Figs in S1 File) [36].

The results in Fig 1 for Wave-V latency with the 40-dB click are similar to the results obtained for several other ABR measures. When the subjects were pooled over race (top panels), the mean latency for females was significantly shorter than for the males (effect size = -1.16; p < 0.0001; in fact, *none* of the 20,000 resampled effect sizes for this comparison exceeded the actually obtained effect size). When the subjects were partitioned by race (bottom panels), female latencies remained shorter than male latencies, but race and sex *interacted*. Namely, the mean latencies for males and females were more similar for the Non-White subjects (effect size = -0.74; p = 0.006) than for the White subjects (effect size = -2.03; p < 0.0001). Interactions of this sort were present for the majority of the ABR measures. (The signs of these effect sizes are negative because, by tradition, the numerator for effect-size calculations for sex difference always was female minus male.) Also evident in Fig 1 is the existence of only a small difference across the menstrual cycle for both race groups, also true for the other ABR measures.

#### 3. Remainder of ABR measures

The effect sizes shown in Table 1 summarize the various comparisons across sex, race, and menstrual cycle for all 8 ABR measures. For this table, the male data used were for "all males" (the Post-VMm and the Pre-VMm males pooled). The *first three rows* of Table 1 show the effect sizes for sex difference using the female data pooled across the menstrual cycle (the average of the two cycle phases) compared with the all-male data, and our discussion will emphasize those results. The following two groups of three rows each (shown for consistency with our previous reports) show the sex differences when the female Menses data are compared with the all-male data, and when the female Midluteal data are compared with the all-male data, respectively. The bottom half of Table 1 shows effect sizes for race difference and menstrual-cycle difference for different pairs of subject groups. The Ns on which these various comparisons were based were similar to the Ns shown in Fig 1; specific values can be found in the supplemental figures [36]. Throughout Table 1, and all subsequent tables, comparisons achieving implied significance are in bold font and superscripted.

**Table 1:** Effect sizes for various pairwise comparisons, shown separately for 8 ABR measures.

			(1)	(2)	(3)	(4)	(5)	(6)	(7)	(8)
			Wave I	Wave I	Wave V	Wave V	Wave V	Wave V	Ratio	Interval
Comparison	Numerator for effect size	Race	Lat. 70 dB	Ampl. 70 dB	Lat. 70 dB	Ampl. 70 dB	Lat. 40 dB	Ampl. 40 dB	V/I 70 dB	I->V 70 dB
Sex difference	Females ignoring cycle^**a**^	Pooled	-0.23	**0.45**^**d**^	**-1.18**^**g**^	**0.32**^**c**^	**-1.16**^**g**^	**0.57**^**e**^	-0.20	**-0.96**^**g**^
	minus all males^**b**^									
		Non-White	-0.12	0.40	**-0.96**^**f**^	0.21	**-0.74**^**e**^	**0.72**^**e**^	-0.25	**-0.83**^**e**^
		White	-0.30	**0.70**^**d**^	**-1.82**^**g**^	**0.81**^**d**^	**-2.03**^**g**^	**0.74**^**d**^	-0.15	**-1.62**^**f**^
	Menses minus all males	Pooled	-0.20	**0.48**^**d**^	**-1.14**^**g**^	**0.38**^**e**^	**-1.25**^**g**^	**0.59**^**e**^	-0.18	**-0.91**^**g**^
		Non-White	-0.14	**0.46**^**e**^	**-0.99**^**f**^	0.27	**-0.86**^**e**^	**0.69**^**e**^	-0.24	**-0.82**^**e**^
		White	-0.16	**0.64**^**e**^	**-1.61**^**f**^	**0.88**^**d**^	**-2.02**^**g**^	**0.88**^**d**^	-0.09	**-1.45**^**f**^
	Midluteal minus all males	Pooled	-0.17	**0.40**^**d**^	**-1.20**^**g**^	0.27	**-1.11**^**g**^	**0.50**^**d**^	-0.26	**-1.10**^**g**^
		Non-White	-0.02	0.28	**-0.99**^**f**^	0.15	**-0.70**^**d**^	**0.67**^**d**^	-0.29	**-1.03**^**f**^
		White	-0.27	**0.74**^**d**^	**-1.80**^**g**^	**0.77**^**d**^	**-1.96**^**g**^	**0.65**^**e**^	-0.24	**-1.65**^**g**^
Race difference	Non-White minus White	All females, ignoring cycle	0.34	-0.32	0.47	-0.55	0.44	0.23	0.08	0.22
		Menses females	0.18	-0.20	0.21	-0.54	0.32	0.13	0.02	0.08
		Midluteal females	0.46	-0.60	0.53	-0.53	0.37	0.21	0.13	0.21
		Males, all	0.24	-0.09	-0.24	0.04	**-0.55**^c^	0.16	0.17	-0.42
Cycle difference	Menses minus midluteal	Pooled	-0.00	-0.06	-0.06	0.05	-0.03	-0.01	0.08	-0.04
		Non-White	-0.07	0.05	-0.18	0.16	-0.09	0.02	0.10	-0.11
		White	0.12	-0.23	0.25	-0.20	0.15	-0.06	0.04	0.17

^a^ Female data averaged across Menses and Midluteal phases of cycle.

^b^ All males = Post-VMm + Pre-VMm males pooled, where VMm = malfunction of voltmeter.

^c^ 0.05 < p < 0.10; implied significance, from resampling.

^d^ 0.01 < p < 0.05.

^e^ 0.001 < p < 0.01.

^f^ 0.0001 < p < 0.001.

^g^ p < 0.0001.

Columns 2–6 and 8 of Table 1 contain generally large effect sizes for sex difference, most of which carry small *p*-values for implied significance (for simplicity we concentrate on the top three rows). The absence of a significant sex difference for latency of Wave I is a common finding e.g., [3, 4, 6, 7, but see 5]. (Even so, note that the direction of the difference was consistent with the other measures.) The significant sex difference for amplitude of Wave I seen in Table 1 also was reported by [6]. The large sex differences for latency and amplitude for Wave V are in accord with numerous past findings (e.g., [3–7]). However, the substantial magnitudes of several of those differences deserve emphasis. Also note that the magnitude of the sex difference was larger for the latency of Wave V than for its amplitude.

Along with Wave-I latency, the amplitude ratio V/I was an unusual ABR measure in that it did not show a substantial sex difference. The V/I ratio has clinical interest because it can be informative about the site-of-lesion in the auditory chain [3].

The large effect sizes for the interpeak interval I–> V (for both races) are in accord with [7, 43], neither of whom partitioned their data by race (also see [3–5]). (Note that this interval difference may be simply the arithmetic consequence of the large sex difference in Wave-V latency.)

Also notable in Table 1 is that the sexes generally were less different for our Non-White subjects than for our White subjects. For 6 of the 8 ABR measures, the effect size for sex difference for the Non-White subjects was about half that for the White subjects. [The two exceptions were columns (6) and (7), Wave-V amplitude with the 40-dB click, and Wave V/Wave I amplitude ratio with the 70-dB click, for which the effect sizes for sex difference were about equal for the two race groups.]

A point deserving emphasis is that although the entries in the middle section of Table 1 reveal that none of the effect sizes for race achieved implied significance for any of the ABR measures, those comparisons all were *within*-sex, so they miss the *interactions* between race and sex that are revealed by examination of the figures. The effect of race is visually compelling in several of the figures made for the individual ABR measures (see S1–S7 Figs in S1 File [36]), just as it is in Fig 1, where the sex difference for the Non-White subjects is noticeably smaller than that for the White subjects.

### B. AMLRs: Sex, race, and menstrual-cycle differences

#### 1. Overview

For AMLRs (as for ABRs), the discussion here focuses on the sex, race, and menstrual-cycle *differences* for the subjects tested; the actual values of the various AMLR latencies and amplitudes measured can be found in (S8-S19 Figs in S1 File) [36].

About half the AMLR latency and amplitude measures exhibited notable sex differences (compare [6, 14]). Sometimes the sex differences were present for both race groups, and sometimes for neither race group. Like the ABR measures, none of the AMLR measures exhibited consistently large differences by race or phase of the menstrual cycle; however, also like the ABR measures, an interaction between race and sex often was visually evident in the figures.

Past findings suggest that both the latency and amplitude of AMLR peaks are more highly dependent upon the specifics of the stimulus than are ABR peaks [14], meaning that the AMLR results reported here must be interpreted as highly parameter-dependent. They may not generalize widely.

We consider the latency and amplitude measures for AMLR in separate sections.

#### 2. AMLR latency

**Fig 2:**
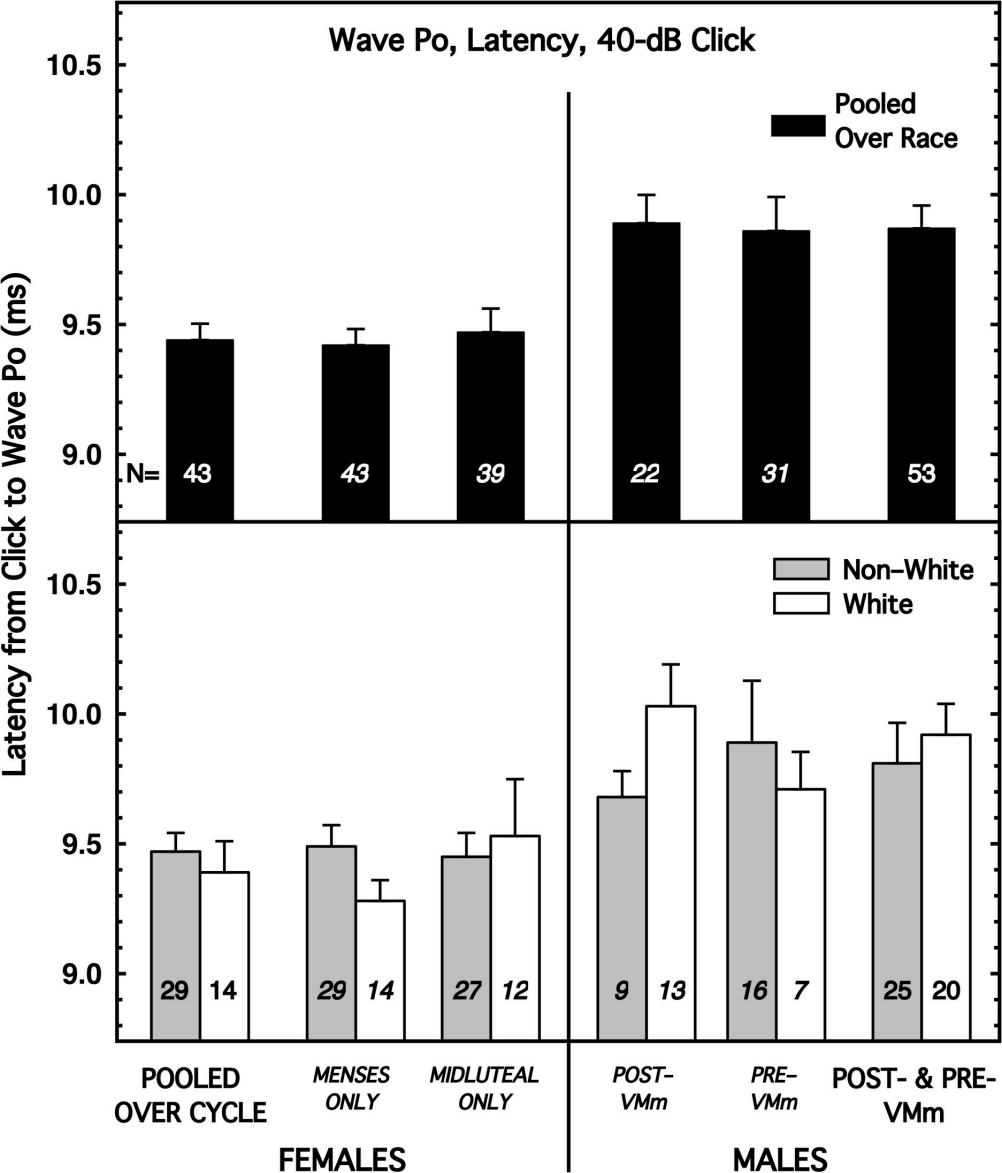
Mean latency for Wave Po with the 40-dB click, shown for females on the left and males on the right. The female data are shown pooled over the menstrual cycle (far left; abscissa label not italicized) and also partitioned by cycle. The male data of most interest are at the far right (abscissa label not italicized), but, for consistency, data also are shown separately for the male subjects tested before and after the malfunction of the voltmeter (VM; which was not used for the ABR or AMLR measurements). (*top*) Subjects are pooled over race. (*bottom*) Subjects are partitioned by race. Flags denote standard errors of the mean. Additional AMLR latency measures are illustrated in (S8-S14 Figs in S1 File) [36].

Fig 2 shows the results for an AMLR latency measure that exhibited large sex differences for both race groups: Wave-Po latency with the 40-dB click. This measure displays the same pattern of results as seen for Wave V in Fig 1. Namely, when the subjects were pooled over race (top panel), the mean latency for females was significantly shorter than for the males (effect size = -0.78; p = 0.0001), and when the subjects were partitioned by race (bottom panel), the sex differences persisted but the mean latencies for males and females were more similar for the Non-White subjects (effect size = -0.57; p = 0.04) than for the White subjects (effect size = -1.05; p = 0.006). This interaction between race and sex is clearly visible in the bottom panels of Fig 2 by comparing the bars at the far left of the female panel *vs*. the bars at the far right of the male panel. Also evident in Fig 2 is that the mean latencies for the two race groups ordered differently for the Post-VMm and Pre-VMm males; this pattern existed for 3 of the 8 AMLR latency measures. The supplementary materials contain figures for each of the other AMLR latency measures (see S8-S14 Figs in S1 File [36]).

The effect sizes for sex, race, and menstrual cycle are shown for each of the 8 AMLR *latency* measures in Table 2, which has the same format as Table 1. As can be seen at the top of Table 2, the sex differences for 4 of the 8 latency measures achieved some level of implied significance (bold font). Except for the measure in column (1), significant sex differences were smaller for the Non-White subjects than for the White subjects, in accord with the pattern for the ABR measures (Table 1). Also notable is that the sex differences for the later waves of the AMLR tended to be larger for the weaker click than for the stronger click. Finally, and also like the ABR results, none of the comparisons for race or cycle differences achieved implied significance, but again, that race comparison is within-sex and thereby misses any *interactions* between race and sex. The moderately large sex difference for Wave Pa for the White subjects is in accord with the report by [14]. McFadden and Champlin [6] reported no significant sex differences for their AMLR measures, for subjects that were pooled across race.

**Table 2:** Effect sizes for various pairwise comparisons, shown separately for 8 AMLR latency measures.

			(1)	(2)	(3)	(4)	(5)	(6)	(7)	(8)
			Wave Po	Wave Po	Wave Na	Wave Na	Wave Pa	Wave Pa	Wave Nb	Wave Nb
Comparison	Numerator for effect size	Race	Lat. 70 dB	Lat. 40 dB	Lat. 70 dB	Lat. 40 dB	Lat. 70 dB	Lat. 40 dB	Lat. 70 dB	Lat. 40 dB
Sex difference	Females ignoring cycle^**a**^	Pooled	**-0.82**^**f**^	**-0.78**^**f**^	-0.00	-0.23	0.00	**-0.36**^**c**^	-0.31	**-0.57**^**e**^
	minus all males^**b**^									
		Non-White	**-0.91**^**f**^	**-0.57**^**d**^	-0.01	-0.39	0.30	0.02	-0.11	-0.25
		White	**-0.86**^**d**^	**-1.05**^**e**^	0.20	-0.05	-0.31	**-0.72**^**d**^	-0.47	**-0.75**^**d**^
	Menses minus all males	Pooled	**-0.79**^**f**^	**-0.82**^**f**^	0.00	-0.09	-0.08	**-0.37**^**c**^	-0.20	**-0.66**^**e**^
		Non-White	**-0.88**^**e**^	**-0.52**^**c**^	0.05	-0.22	0.24	-0.02	0.11	-0.34
		White	**-0.79**^**d**^	**-1.42**^**f**^	0.10	0.06	-0.42	**-0.71**^**c**^	-0.54	**-0.90**^**d**^
	Midluteal minus all males	Pooled	**-0.84**^**f**^	**-0.65**^**e**^	-0.07	**-0.35**^**c**^	-0.14	-0.31	**-0.51**^**d**^	**-0.60**^**e**^
		Non-White	**-0.91**^**e**^	**-0.57**^**d**^	-0.14	**-0.54**^**c**^	0.16	0.10	-0.26	-0.23
		White	**-0.89**^**d**^	**-0.62**^**c**^	0.19	-0.07	-0.44	**-0.70**^**c**^	**-0.84**^**d**^	**-0.94**^**d**^
Race difference	Non-White minus White	All females, ignoring cycle	0.32	0.18	-0.24	0.06	0.38	0.42	0.31	0.33
		Menses females	0.13	0.53	-0.02	0.08	0.43	0.34	0.57	0.33
		Midluteal females	0.42	-0.14	-0.32	-0.10	0.36	0.49	0.51	0.47
		Males, all	0.01	-0.16	0.03	0.33	-0.22	-0.33	-0.02	-0.31
Cycle difference	Menses minus midluteal	Pooled	0.09	-0.10	0.09	0.32	0.02	-0.08	0.05	-0.17
		Non-White	0.01	0.10	0.21	0.35	0.01	-0.09	0.16	-0.21
		White	0.24	-0.48	-0.18	0.24	0.04	-0.04	-0.22	-0.08

^a^ Female data averaged across Menses and Midluteal phases of cycle.

^b^ All males = Post-VMm + Pre-VMm males pooled, where VMm = malfunction of voltmeter.

^c^ 0.05 < p < 0.10; implied significance, from resampling.

^d^ 0.01 < p < 0.05.

^e^ 0.001 < p < 0.01.

^f^ 0.0001 < p < 0.001.

^g^ p < 0.0001.

#### 3. AMLR amplitude

The amplitude measures for AMLR showed fewer notable differences between the sexes than did the amplitude measures for ABR discussed above. That said, these data did exhibit a feature contrary to one seen for most of the ABR and AMLR latency measures discussed so far: for all 6 measures of AMLR amplitude, the effect sizes for sex difference were numerically larger for the *Non-White* subjects than for the White subjects.

**Fig 3:**
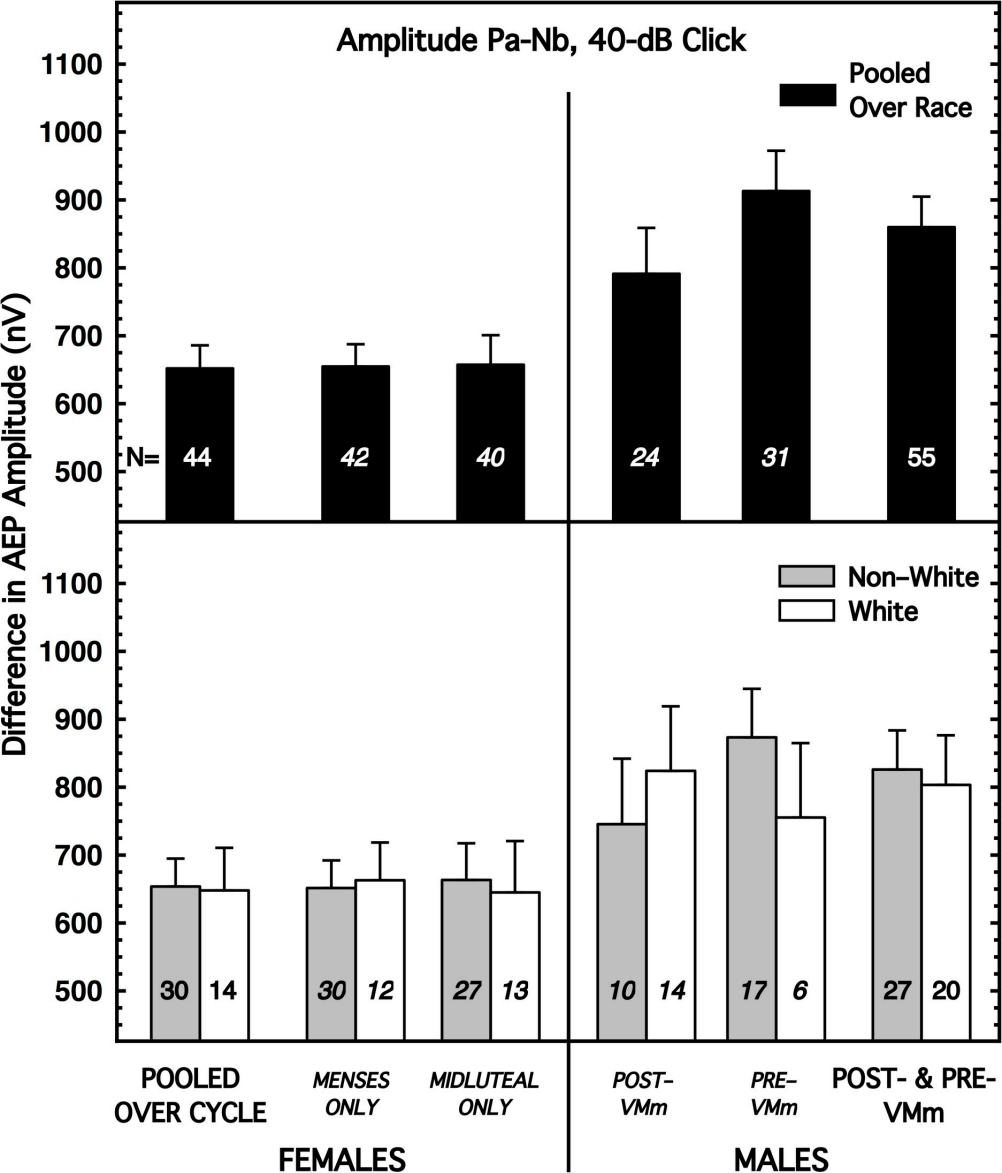
Mean amplitude for Wave Pa-Nb with the 40-dB click, shown for females on the left and males on the right. The female data are shown pooled over the menstrual cycle (far left; abscissa label not italicized) and also partitioned by cycle. The male data of most interest are at the far right (abscissa label not italicized), but, for consistency, data also are shown separately for the male subjects tested before and after the malfunction of the voltmeter (VM; which was not used for the ABR or AMLR measurements). (*top*) Subjects are pooled over race. (*bottom*) Subjects are partitioned by race. Flags denote standard errors of the mean. Additional AMLR amplitude measures are illustrated in (S15-S19 Figs in S1 File) [36].

The AMLR amplitude measure showing the largest sex difference and a modest interaction between sex and race (Pa-Nb amplitude with the 40-dB click) is shown in Fig 3. As before, the bars of most interest are those at the far left and far right of the upper and lower panels. The differences by sex, race, and menstrual cycle for all 6 AMLR measures of amplitude are summarized in Table 3, and the details of Fig 3 and Table 3 are discussed in section IIIsupp.B.3 in S1 File [36].

**Table 3:** Effect sizes for various pairwise comparisons, shown separately for 6 AMLR amplitude measures.

			(1)	(2)	(3)	(4)	(5)	(6)
			Po-Na	Po-Na	Na-Pa	Na-Pa	Pa-Nb	Pa-Nb
Comparison	Numerator for effect size	Race	Ampl. 70 dB	Ampl. 40 dB	Ampl. 70 dB	Ampl. 40 dB	Ampl. 70 dB	Ampl. 40 dB
Sex difference	Females ignoring cycle^**a**^	Pooled	0.30	0.27	-0.16	**-0.36**^**c**^	**-0.47**^**d**^	**-0.72**^**f**^
	minus all males^**b**^							
		Non-White	0.35	0.34	-0.15	-0.34	**-0.51**^**c**^	**-0.66**^**d**^
		White	0.17	0.07	-0.14	-0.31	-0.35	-0.53
	Menses minus all males	Pooled	**0.34**^**c**^	0.32	-0.11	-0.31	**-0.36**^**c**^	**-0.71**^**e**^
		Non-White	0.41	0.35	-0.10	-0.33	-0.36	**-0.67**^**d**^
		White	0.17	0.21	-0.10	-0.15	-0.34	-0.50
	Midluteal minus all males	Pooled	0.27	0.10	-0.16	-0.32	**-0.40**^**c**^	**-0.65**^**e**^
		Non-White	0.36	0.26	-0.17	-0.28	**-0.49**^**c**^	**-0.56**^**d**^
		White	0.05	-0.29	-0.13	-0.29	-0.13	-0.52
Race difference	Non-White minus White	All females,	0.15	0.26	0.05	0.05	-0.12	0.02
		ignoring cycle						
		Menses females	0.22	0.12	0.05	-0.14	0.09	-0.05
		Midluteal females	0.27	0.53	0.03	0.07	-0.41	0.07
		Males, all	-0.03	-0.02	0.05	0.06	0.11	0.07
Cycle difference	Menses minus midluteal	Pooled	0.12	0.28	0.02	-0.06	0.09	-0.00
		Non-White	0.19	0.17	0.15	-0.02	0.18	-0.02
		White	-0.02	0.62	-0.17	-0.14	-0.11	0.04

^a^ Female data averaged across Menses and Midluteal phases of cycle.

^b^ All males = Post-VMm + Pre-VMm males pooled, where VMm = malfunction of voltmeter.

^c^ 0.05 < p < 0.10; implied significance, from resampling.

^d^ 0.01 < p < 0.05.

^e^ 0.001 < p < 0.01.

^f^ 0.0001 < p < 0.001.

^g^ p < 0.0001.

### C. Averaged waveforms

As noted above, averaged waveforms were calculated for each subject group as a way of illustrating the sex, race, and menstrual-cycle differences detailed in the figures and tables above. Although averaged waveforms have appeal as qualitative depictions of AEP data, they possess several weaknesses (discussed in section IIsupp.G.2 in S1 File [36]), one of which is illustrated below. The authors regard the peak-by-peak analyses presented in the above figures and tables to be preferable to averaged waveforms for summarizing AEP data, and for hypothesis testing. With that caveat, averaged waveforms are presented here and in the online (S1 File) for each of the relevant comparisons. For these analyses, individual waveforms having anomalies for single peaks but usable for other peaks were included in the average in their entirety. This strategy contributed to the small discrepancies between the averaged waveforms and the tabled data above. Neither here nor in the (S1 File) were any constant offsets added to the averaged waveforms to make the pre-click waveforms equal for the two groups under consideration; the high similarity in the averaged waveforms before click presentation was real.

**Fig 4:**
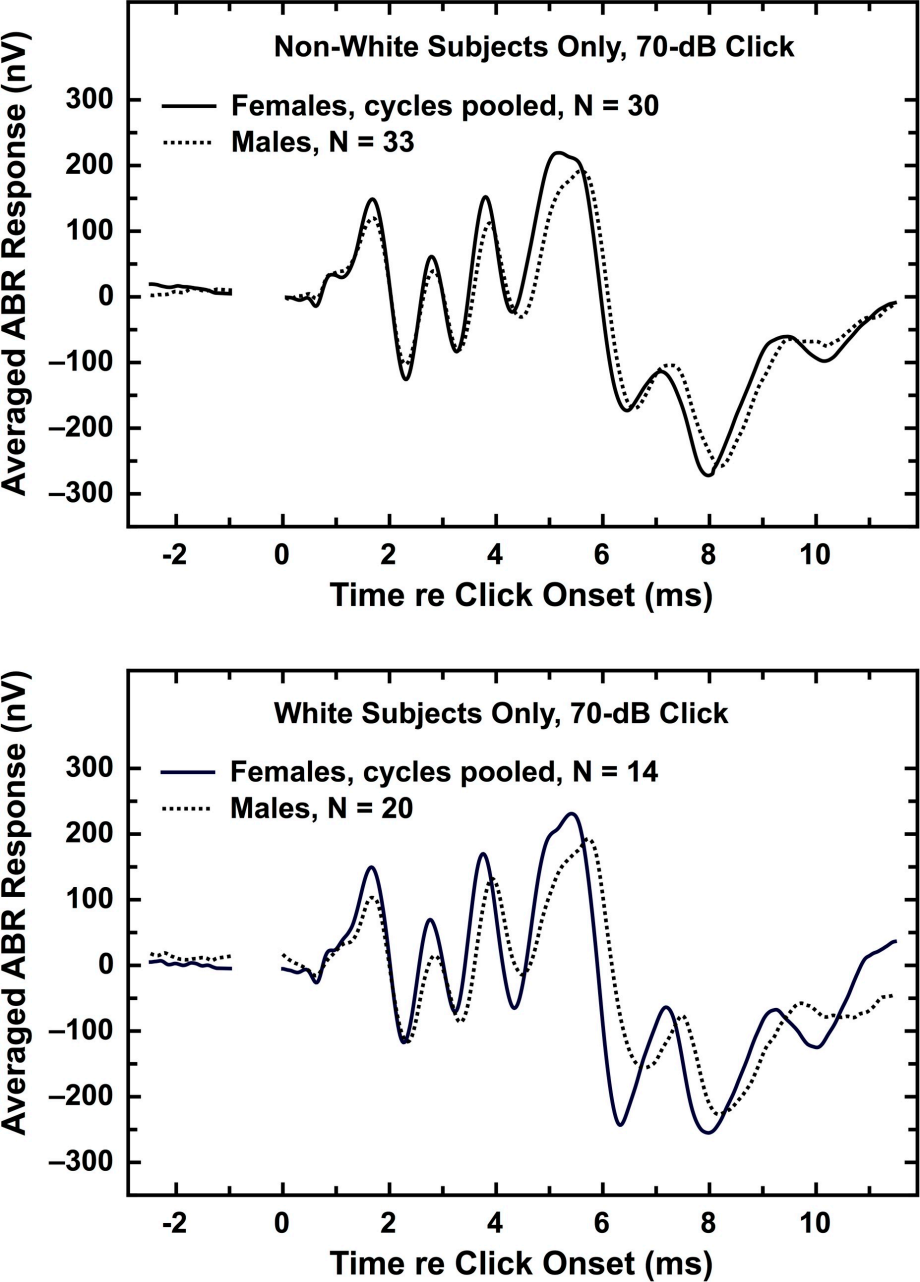
Averaged waveforms illustrating the sex differences for ABR, shown separately for the two race groups, using the 70-dB click. Each panel contains an averaged waveform for females pooled across the menstrual cycle (solid trace) and an averaged waveform for all males (dotted trace). (*top panel*) Non-White females and males. (*bottom panel*) White females and males. A 1-ms segment has been deleted from all waveforms during the baseline period in order to remove an electrical artifact. Additional comparisons of averaged waveforms are shown in (S21-S46 Figs in S1 File) [36].

The top panel of Fig 4 contains the averaged ABR waveforms for Non-White females (menstrual cycle pooled) and Non-White males; the bottom panel contains the same for the White subjects. For the ABR waveforms in Fig 4, and for all subsequent ABR waveforms, no data are shown for a 1-ms interval preceding time zero. The reason is that, even though the earphone was shielded, many of the individual waveforms contained an electrical artifact during this time period. For all the averaged waveforms shown here, time zero marks the arrival of the click stimulus in the ear canal. Visual examination of the waveforms in Fig 4 reveals that female AEPs generally exhibit shorter latencies and stronger amplitudes than male AEPs, just as shown in Fig 1 and Table 1.

Fig 4 also illustrates one of the serious weaknesses of averaged waveforms over peak-by-peak analysis. All the individual waveforms contributing to the four averaged waveforms in Fig 4 were examined for the presence of a Wave IV that was visibly distinct from Wave V. Wave IV was present in 78% of the subjects (see section IIsupp.G.2 in S1 File [36]). Yet none of the four averaged waveforms in Fig 4 exhibits a clear Wave IV. Because characteristics possessed by the majority of the individual subjects can be lost in the averaging process, both authors and readers need to be cautious about any use of averaged waveforms as summaries of AEP results.

**Fig 5:**
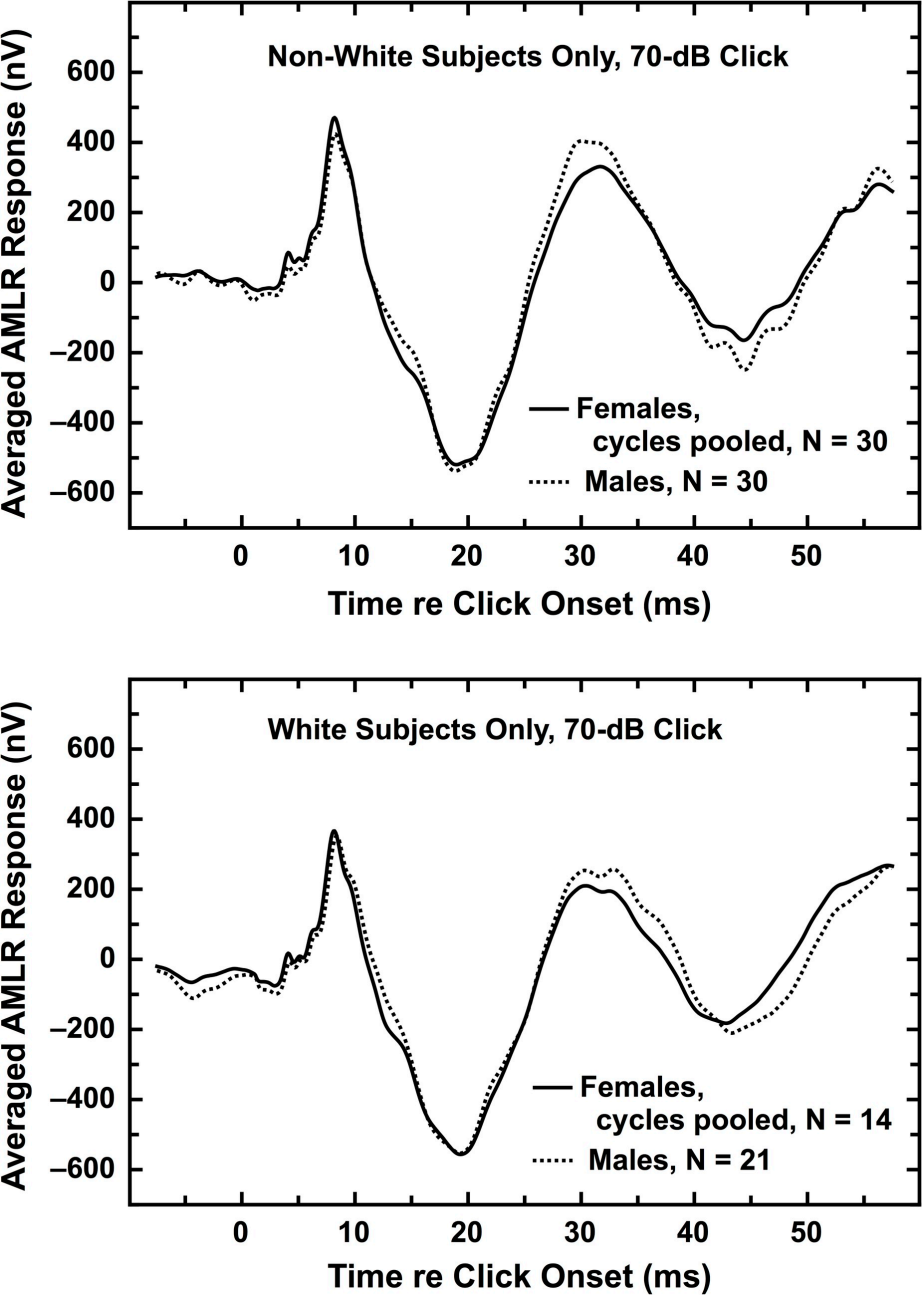
Averaged waveforms illustrating the sex differences for AMLR, shown separately for the two race groups, using the 70-dB click. Each panel contains an averaged waveform for females pooled across the menstrual cycle (solid trace) and an averaged waveform for all males (dotted trace). (*top*) Non-White females and males. (*bottom*) White females and males.

Fig 5 shows averaged waveforms for AMLR in the same format as used in Fig 4. The Non-White groups are in the top panel and the White groups are in the bottom panel.

Several other figures with averaged waveforms summarizing various sex, race, and menstrual-cycle comparisons can be found in (S21-S45 Figs in S1 File) [36].

### D. Correlations between AEP measures

#### 1. ABRs

One obvious question to ask about the AEP measures is how they were correlated with each other. The answers are shown for the ABR measures in Table 4. In the top right half of the table, in italics, are the intercorrelations for females; the intercorrelations for males are at the bottom left of Table 4. For this table, the female data used were for subjects averaged across the phases of the menstrual cycle; the male data used were for subjects pooled over Post-VMm and Pre-VMm (all males). The various rows of Table 4 show the correlations for subjects pooled over race and partitioned by race. Ns for the various measures are shown in (Figs 1–3; S1-S19 Figs in S1 File) [36].

**Table 4:** Correlations between 8 ABR measures, shown separately for females^a^ (upper right, italics) and all-males^b^ (lower left).

		(1)	(2)	(3)	(4)	(5)	(6)	(7)	(8)
		Wave I,	Wave I,	Wave V,	Wave V,	Wave V,	Wave V,	Wave V/Wave I,	Wave I -> V
ABR measure	Race	Lat., 70 dB	Ampl., 70 dB	Lat., 70 dB	Ampl, 70 dB	Lat., 40 dB	Ampl., 40 dB	Ratio, 70 dB	Interval, 70 dB
Wave I latency,	Pooled	- - -	*-0*.*23*	*0*.*09*	*0*.*02*	*0*.*02*	*-0*.*03*	***0*.*42*** ^c^	***-0*.*46*** ^d^
70 dB	Non-White	- - -	*-0*.*26*	*-0*.*09*	*0*.*33*	*-0*.*17*	*0*.*09*	***0*.*62*** ^d^	***-0*.*55*** ^c^
	White	- - -	*-0*.*09*	*0*.*44*	*-0*.*43*	*0*.*42*	*-0*.*37*	*-0*.*29*	*-0*.*29*
Wave I amplitude,	Pooled	**-0.41**^e^	- - -	*-0*.*22*	*0*.*21*	***-0*.*36*** ^c^	*0*.*29*	***-0*.*69*** ^f^	*-0*.*06*
70 dB	Non-White	**-0.50**^d^	- - -	*-0*.*21*	*0*.*15*	*-0*.*38*	*0*.*28*	***-0*.*74*** ^e^	*-0*.*04*
	White	-0.08	- - -	*-0*.*11*	*0*.*26*	*-0*.*19*	*0*.*40*	***-0*.*51*** ^c^	*-0*.*05*
Wave V latency,	Pooled	0.21	**-0.48**^e^	- - -	***-0*.*47*** ^d^	***0*.*61*** ^e^	***-0*.*40*** ^c^	*-0*.*06*	***0*.*84*** ^g^
70 dB	Non-White	**0.50**^d^	**-0.51**^d^	- - -	*-0*.*46*	***0*.*65*** ^d^	***-0*.*62*** ^d^	*-0*.*04*	***0*.*88*** ^f^
	White	0.12	**-0.41**^c^	- - -	*-0*.*41*	*0*.*41*	*0*.*06*	*-0*.*19*	***0*.*73*** ^e^
Wave V amplitude,	Pooled	-0.17	**0.34**^d^	**-0.30**^d^	- - -	***-0*.*51*** ^d^	***0*.*52*** ^d^	***0*.*46*** ^d^	***-0*.*42*** ^d^
70 dB	Non-White	-0.15	0.35	-0.23	- - -	*-0*.*48*	***0*.*62*** ^d^	*0*.*45*	***-0*.*54*** ^c^
	White	-0.06	0.02	**-0.39**^c^	- - -	***-0*.*52*** ^c^	***0*.*48*** ^c^	***0*.*66*** ^d^	*-0*.*11*
Wave V latency,	Pooled	**0.40**^e^	**-0.42**^e^	**0.54**^f^	-0.23	- - -	***-0*.*36*** ^c^	*-0*.*01*	***0*.*54*** ^d^
40 dB	Non-White	0.33	**-0.42**^c^	**0.69**^f^	-0.23	- - -	*-0*.*40*	*0*.*06*	***0*.*62*** ^d^
	White	**0.51**^d^	**-0.46**^d^	**0.43**^c^	-0.17	- - -	*-0*.*39*	*-0*.*34*	*0*.*12*
Wave V amplitude,	Pooled	-0.10	0.08	-0.09	**0.45**^e^	0.01	- - -	*0*.*07*	*-0*.*33*
40 dB	Non-White	**-0.43**^c^	0.08	-0.14	**0.49**^d^	-0.25	- - -	*0*.*05*	***-0*.*55*** ^c^
	White	0.24	-0.13	0.09	**0.43**^c^	0.30	- - -	*0*.*13*	*0*.*35*
Wave V/Wave I ratio	Pooled	0.22	**-0.61**^f^	0.22	**0.38**^d^	0.17	0.19	- - -	*-0*.*28*
70 dB	Non-White	0.27	**-0.64**^e^	0.32	0.37	0.18	0.21	- - -	*-0*.*34*
	White	0.15	**-0.67**^e^	0.05	**0.57**^d^	0.28	0.36	- - -	*0*.*02*
Wave I -> V interval,	Pooled	**-0.35**^d^	-0.18	**0.84**^g^	-0.20	0.21	0.00	-0.05	- - -
70 dB	Non-White	-0.08	-0.16	**0.83**^f^	-0.14	**0.50**^d^	0.23	-0.04	- - -
	White	-0.35	-0.33	**0.89**^g^	**-0.44**^c^	0.04	-0.11	-0.10	- - -

^a^ Female data averaged across Menses and Midluteal phases of cycle.

^b^ All males = Post-VMm + Pre-VMm males pooled, where VMm = malfunction of voltmeter.

^c^ 0.05 < p < 0.10; implied significance, from resampling.

^d^ 0.01 < p < 0.05.

^e^ 0.001 < p < 0.01.

^f^ 0.0001 < p < 0.001.

^g^ p < 0.0001.

Generalizations are difficult to extract from the correlations in Table 4. Overall, the correlations do not appear to be higher for one sex over the other, nor higher for one race group over the other. Similarly, there is no compelling tendency for latency measures to correlate better with other latency measures than with amplitude measures, nor the converse. Some specific outcomes are discussed in section IIIsupp.D.1 in S1 File [36].

#### 2. AMLRs

Intercorrelations like those in Table 4 also were calculated for the AMLR measures. The interrelationships between the 8 AMLR latency measures are shown in (S1 Table in S1 File) [36], and the interrelationships for the 6 AMLR amplitude measures are shown in (S2 Table in S1 File). Those outcomes are discussed in section IIIsupp.D.2 in S1 File [36]. To summarize those results, there were few high correlations, and the patterns of results across sex and race were not similar for the latency and amplitude measures.

#### 3. Other correlations

Also calculated were the correlations between the various ABR measures and the various AMLR measures (see S3-S8 Tables and section IIIsupp.D.3 in S1 File [36]). In general, the patterns of results were not consistent across sex or race.

Also calculated were the correlations between the various AEP measures and the various OAE measures (see S9-S11 Tables and section IIIsupp.D.3 in S1 File [36]). These correlations were weak for both sexes, both within and across race groups, and the correlations between OAEs and the ABR waves were not stronger than those between OAEs and the AMLR waves.

Also calculated were the correlations between the various AEP measures and the various psychoacoustical tasks (see S12-S17 Tables and section IIIsupp.D.3 in S1 File [36]). The outcome was that no correlations between individual AEP measures and the various psychoacoustical tasks were large and consistent across the race groups or across sexes.

## IV. Discussion

### A. Sex differences

#### 1. Summary

Some sex differences previously reported for AEPs were confirmed. Also confirmed were previous reports of the absence of sex differences for specific AEP measures. For ABR measures, latency generally was shorter and amplitude larger for females than for males, in accord with past findings; for some AMLR measures that directionality was reversed. The sizes of some of our sex differences were quite substantial (see Table 1 and Fig 1, also [36]). These findings address goal 1 in section I.A.

#### 2. Specifics

Three generalizations can be made about the sex differences observed here for the various AEP measures. First, sex differences were larger and more numerous for ABR measures than for AMLR measures. Second, sex differences were more numerous and larger for the latency measures than for the amplitude measures and this was true for both ABRs and AMLRs. Third (and this was a weaker trend), for a particular AEP measure, there was a tendency for the sex difference to be larger for the weak click than for the stronger click.

The largest sex differences observed were for Wave-V latency. For both click levels, those effect sizes were about -1.2 (shorter latency for females than males); also for both click levels, *none* of the 20,000 random resamples yielded an effect size larger than the value actually obtained. The next largest sex differences were for Wave-Po latency. For both click levels, those effect sizes were about -0.8, with implied significance of 0.0001 from resampling. The latency measures involving Waves Na or Pa yielded no sex difference that was large, medium, or significant. For amplitude measures, the largest sex difference was for the last wave measured, Pa-Nb with the 40-dB click. That effect size was -0.72 (*smaller* amplitude for females than for males) and the implied significance was 0.0006.

In accord with past findings [3, 5–7, 43], the sex difference for Wave-I latency was small and not significant under resampling. However, the sex difference for Wave-I amplitude was medium and did achieve implied significance (p = 0.02) (also seen in [6]). One interpretation of this outcome is that the basis for the sex difference in latency (in Wave V and beyond) may not reside solely in the cochlea; see [6].

Also in accord with past findings [3–5], the sex difference for the interpeak interval I->V was large. McFadden et al. [43] searched for sex differences in the time intervals between all pairs of successive peaks in the AEP (ABRs, AMLRs, and ALRs). For AMLRs, no sex difference achieved implied significance, but for ABRs, the interpeak interval I->V did exhibit a significant sex difference, whichever ear was stimulated.

For readers interested in the physiological origins of sex differences in ABRs and AMLRs, note that [6] reported that several ABR measures showing sex differences also differed for heterosexual and non-heterosexual subjects.

### B. Race differences

#### 1. Summary

Many of the sex differences for AEPs were of different magnitudes for the two race groups, generally larger for the White subjects. When race differences were compared *within-sex* (Tables 1–3), those race differences never achieved implied significance. Still, the *interaction* of race and sex was readily evident in graphs of the latencies and amplitudes of various AEP waves. As previously reported for these same subjects, the effect sizes for sex difference also were smaller for Non-Whites than for Whites for two out of three measures of OAEs [2], for 6 out of 7 psychoacoustical tasks [1], and for a two-tone masking task known as the Greenwood effect [28]. These findings address goals 1 and 4 in section I.A.

#### 2. Specifics

Prior to this report, race differences had been reported in the auditory periphery (OAEs) and the auditory behavior of these same subjects [1, 2]. Now we have shown that race differences also can be seen in AEPs, measures of the auditory chain lying between the OAEs in the periphery and wherever in the brain auditory behavior begins. Although our subjects did not exhibit race differences within-sex, but only as interactions, other samples of subjects, with different racial make-ups, might show differences within-sex as well as differences in the size of the sex differences. For example, the data in [5] contain within-sex race differences (see goal 4, section I.A).

All the information we had about the race/ethnicity of our subjects came from the two questions required by the National Institutes of Health (NIH; see section IIsupp.A in S1 File [36]). Those items pool manifestly different race groups (e.g., “Asian” pools East Asians with Southeast Asians) and as a consequence, both of our race groups surely were heterogeneous regarding the factor(s) underlying the differences between our race groups. Replications of this research, with more precise categories of race, are necessary before anyone can know how race groups truly differ on the measures reported here.

#### 3. Two comments

Updating and expanding the NIH items on race/ethnicity, and developing additional measures of race/ethnicity, would be a benefit to investigators curious about the extent and magnitude of race differences in audition and all the other research disciplines covered by NIH funding. Elsewhere [1, 2], we have argued that, for cochlear measures, race categories are likely to be a (poor) proxy for the actual variable of interest (perhaps, density of intracochlear melanocytes). For AEPs (and various non-auditory measures), it is not presently clear what the variable(s) underlying race differences are, but we continue to believe that future discoveries will render race an unnecessary intermediate proxy for the factors actually relevant to the individual differences correlated with “race” [44]. Differences observed across race categories are not *explained* by "race"; the differences necessarily arise from basic cellular mechanisms that happen to be correlated with race categories. Similarly, any auditory *sex* difference ultimately will be attributable to some cellular, metabolic, or hormonal difference that is correlated with sex. Eventually, "race" and "sex" will not be *the explanations* for the underlying differences; they will be proxies for them.

Because the present study was not designed with race in mind, many obvious comparisons cannot be made, and the outcomes obtained must be interpreted only as suggestive until truly experimental tests are implemented. A point that should not have to be made is: one race group having larger effect sizes or correlations, or having more effect sizes or correlations that achieve implied significance, does not make that race group “better” than some other race group. These are simply facts about the samples of subjects measured here that might be replicated in future studies. *Differences must not be interpreted as deficiencies*. There is no apparent real-world advantage to having larger or more-numerous significant effect sizes or correlations, and the actual differences in stimulus units are so small they likely are immaterial to everyday perception. To be sure, if these outcomes replicate, scientists will want to understand the mechanisms underlying the differences, and in the process, they might learn something important about individual differences in the fundamental mechanisms of hearing. However, whatever is learned would not position one race group above another.

Although race itself is only a (temporary) proxy variable, we hope interest in race increases because finding auditory race differences is a step in the direction of finding those individual differences in biology and physiology that underlie the basic mechanisms of everyday hearing.

### C. Menstrual-cycle differences

#### 1. Summary

Contrary to expectation, all of the comparisons between the Menses and Midluteal phases of the menstrual cycle exhibited small effect sizes and none achieved implied significance (see Tables 1–3). These findings address goals 2 and 4 in section I.A.

#### 2. Specifics

When designing this study, the expectation was that we would confirm previous reports about menstrual effects on various auditory measures e.g., [15–21] and possibly add additional measures to that list. Thus, the absence of any notable effects of the menstrual cycle was a surprise. This study used a superior method for identifying the phases of each individual subject’s cycle (see section IIsupp.B.1 in S1 File [36]), only naturally-cycling women were employed, and, for most subjects, auditory measures were obtained during multiple cycles, three strengths of the study that were expected to yield strong evidence of the expected menstrual effects. Instead we have failures to replicate, but failures need to be reported [29, 30]. For readers wondering if our measures of latency and amplitude might miss some menstrual-cycle difference, the averaged waveforms for the Menses and Midluteal phases were very nearly superimposed. One point that may prove crucial is that all our comparisons were between menses and midluteal phases whereas some previous reports compared menses and ovulatory phases. Other possible explanations are considered in section IVsupp.C.2 in S1 File [36]. Circumstantial evidence that cyclic hormonal fluctuations ought to affect AEPs and OAEs is that use of oral contraceptives does affect both physiological measures [23].

### D. Lack of level effect

The amplitude and latency for numerous physiological measures, including AEPs, are known to vary with stimulus magnitude (e.g., [3, 45–49]). As the stimulus strengthens, response amplitude typically increases and latency decreases. Those common findings generally were observed here, but they were weak or absent for two measures involving Wave Pa, especially for the females. Details are provided in section IVsupp.D in S1 File [36].

### E. Correlations between AEPs, and between AEPs and OAEs

Correlations were calculated to determine how the various AEP measures were related to each other, and how the various AEP measures were related to OAE and psychoacoustical measures obtained from the same subjects. Because sex differences existed for many of the AEP measures, all correlations were done within-sex, both pooled over race and partitioned by race. All possible intercorrelations were performed for the 8 ABR measures, for the 8 AMLR latency measures, and for the 6 AMLR amplitude measures. In addition, all the ABR measures were compared with all the AMLR measures, and all the ABR and AMLR measures were compared with 4 types of OAE. The outcomes were summarized in section III.D. These findings address goals 5 and 6 in section I.A.

Many of the intercorrelations for ABRs and for AMLRs were large and significant. However, most of the correlations calculated between AEPs and OAEs were small and did not achieve implied significance; those that did achieve implied significance typically were not consistent across sex or race (see S9-S11 Tables in S1 File [36]). The implication of the latter outcome is that the individual differences in OAEs do not propagate directly to the neural stages of processing in the auditory system, rather a curious outcome.

### F. Correlations between AEPs and psychoacoustical performance

Correlations also were calculated to determine how the various AEP measures related to performance on the seven psychoacoustical tasks also measured on these same subjects. In general, these correlations were small, did not achieve implied significance, and were not consistent across sex or race groups. That is, our results suggest that the individual differences in AEP measures are not well correlated with the individual differences present in performance for any of the psychoacoustical tasks we studied. This outcome parallels the previous finding that various OAE measures also correlated only weakly with performance on those same psychoacoustical tasks [2, 28]. These findings address goal 3 in section I.A.

As noted above, when planning this study, we had less reason to expect strong correlations between individual AEP measures and performance on any of the psychoacoustical tasks than to expect strong correlations between some of the OAE measures and performance on some of the psychoacoustical tasks. After all, the various AEP measures are neural responses to single sounds that were unmasked, wideband, and impulsive, while the stimuli used for our psychoacoustical tasks typically were not. Also, many of the AEP responses measured here originate from early way-stations in the neural chain between the cochlea and auditory cortex (e.g., [50]), whereas behavioral decisions (surely) arise in the cortex. On the other hand, the idea that AEPs can be revealing about auditory ability has a long history in auditory science e.g., [3, 25, 26], and because the plan was to collect AEPs for other purposes anyway (e.g., to assess sex differences, and for comparisons with OAEs), the comparisons between AEPs and the various psychoacoustical tasks were a fringe benefit. Besides, until the comparisons were made, the pessimistic expectations remained untested.

The absence of even moderate correlations between Wave V and detection of the 3.0-kHz tone in the quiet was a minor surprise (S12 and S13 Tables in S1 File [36]). From the beginning, one of hearing science’s primary motivations for studying AEPs was the search for objective measures of hearing sensitivity, and various studies concluded that Wave V and behavioral thresholds are reasonably correlated (e.g., [3, 51, compare 40, see 26]).

The differences in the stimuli used for AEPs and the psychoacoustical tasks likely contributed to some of the weak correlations between the two sets of measures. That said, the peaks of the ABR, at least, commonly are attributed to neural activity originating from the basal (high-frequency and mid-frequency) region of the basilar membrane [13], where the 3.0-kHz signal used for our psychoacoustical tasks would be maximally effective [3].

One possibility is that our relatively stringent screening of subjects resulted in *small* individual differences in auditory sensitivity (and other psychoacoustical measures) compared to larger individual differences in AEPs, thus producing weak correlations.

Weak correlations can result when there is high variability in the measurements; here we averaged over multiple AEP (and OAE) measurements for each female subject, and, for both sexes, the psychoacoustical means were based on many blocks of trials for each subject (see [1]). Thus, we believe that the precision of measurement here was higher than in many auditory studies. Our test/retest results showed excellent reliability (see section IIsupp.D in S1 File [36]).

### G. Greater male variability

A long-standing question in the scientific discussion of sex differences is whether the distributions of female and male values have different shapes. Specifically, it long has been observed that the variability of the male distribution often is greater than that of the female distribution, meaning that more males exist at the extremes. A review can be found in [52]. Although it was not part of the original research plan, this question was examined for the 22 AEP measures obtained in this study by calculating the ratio (male variance/female variance).

The result was that the variances for males were larger than those for the females for 73% of the AEP measures. The mean sex ratio was about 1.2 for the ABR measures, and about 2.0 and 1.8 for the latency and amplitude measures for AMLR, respectively. Those were the results when the data were pooled over race and menstrual cycle for the females and pooled over race for the males, but the tallies were similar when the data were partitioned by race. Accordingly, the numerous interactions between sex and race reported here were not the result of systematic differences in variance for the two race groups. Generally similar percentages for the variance ratios were obtained when the four OAE and seven psychoacoustical measures were examined in the same way. Thus, we provide modest support for the "greater male variability hypothesis," using measures that are highly unlikely to be subject to social or cultural influences (see [52]).

For completeness we note that the mean/variance ratios for the 22 AEP measures were invariably smaller than 1.0 for the amplitude data and typically larger than 10.0 for the latency data, with no obvious trends by sex or race.

### H. Final comments

It is important to note that negative results are not equivalent to no results. Negative results can contribute to knowledge, if not the same way as positive results. For example, the absence of strong correlations between AEPs and our psychoacoustical tasks suggests that the electrical potentials we recorded from the scalp either are not precise enough to detect activity in the relevant neural channels, or were not evoked by an appropriate stimulus, or some other explanation. Our negative results suggest changes in the experimental procedure that future investigators might explore to find stronger correlations (different arrays of electrodes, different evoking stimuli, etc.) and thereby possibly learn more about processing in the brain. As another example, the relative absence of large sex differences in latency for the later peaks in the AMLR (Table 2 plus tables and figures in [36]) demonstrates that the large sex differences seen for Wave V are not preserved at all subsequent stages of neural processing. That is potentially valuable information about sex differences in brain structure. The absence of an effect can lead to hypotheses about "why," and how to find out.

To our regret, we will not be contributing further to this body of knowledge because this lab now is closed because of retirements.

## Supporting information

S1 File (DOCX)
