# Supplementary material for: Auditory evoked potentials: Differences by sex, race, and menstrual cycle and correlations with common psychoacoustical tasks
Source: PLoS One. 2021 May 12;16(5):e0251363. doi: 10.1371/journal.pone.0251363 (PMC8115856; doi:10.1371/journal.pone.0251363)
Supplement: S1 File — (DOCX) [file pone.0251363.s001.docx]

**This is an ONLINE SUPPLEMENT to the published article**

**McFadden, D., Champlin, C.A., Pho, M.H., Pasanen, E.G., Maloney, M.M., and Leshikar, E.M. "Auditory evoked potentials: Differences by sex, race, and menstrual cycle and correlations with common psychoacoustical tasks," Public Library of Science (PLOS ONE), 2021, in press, *****

**The URL/doi for this online supplement is:**

**https://repositories.lib.utexas.edu/handle/2152/81119 doi.org/10.26153/tsw/8132**

**The purpose of this supplement is to provide details about topics omitted from the published article because of space considerations. The headings and the organization of the text here are identical to that of the published article, but for copyright reasons, the published text has been omitted here and only the supplemental text appears. To make citations to the various sections unambiguous, the major headings are relabeled here with a "supp" (e.g., Isupp. INTRODUCTION, IIsupp. METHOD, etc.)**

**Files containing supplementary tables and figures can be found using the links in the Table of Contents.**

**Table of Contents for Supplementary Materials**

INTRODUCTION Isupp

Experimental objectives Isupp.A

Comment Isupp.B

METHODS IIsupp

Subjects IIsupp.A

General Procedures IIsupp.B

**Menstrual cycle* IIsupp.B.1**

Measuring auditory evoked potentials IIsupp.C

**Measurement details for ABRs and AMLRs IIsupp.D**

**Two complications IIsupp.E**

**Statistical analyses and resampling IIsupp.F**

Averaged waveforms IIsupp.G

Description IIsupp.G.1

**Weaknesses of averaged waveforms IIsupp.G.2**

Strategy IIsupp.H

RESULTS IIIsupp

ABRs: Sex, race, and menstrual-cycle differences IIIsupp.A

Overview IIIsupp.A.1

Representative ABR outcome IIIsupp.A.2

Remainder of ABR measures IIIsupp.A.3

AMLRs: Sex, race, and menstrual-cycle differences IIIsupp.B

Overview IIIsupp.B.1

AMLR latency IIIsupp.B.2

**AMLR amplitude IIIsupp.B.3**

Averaged waveforms IIIsupp.C

Correlations between AEP measures IIIsupp.D

**ABRs IIIsupp.D.1**

**AMLRs IIIsupp.D.2**

**Other correlations IIIsupp.D.3**

**Additional Summary Tables *https://repositories.lib.utexas.edu/handle/2152/69855***

***or http://doi.org/10.15781/T25Q4S580.***

**Intercorrelations, AMLR Table S1 - S2**

**Correlations, ABR vs. AMLR Table S3 - S6**

**Correlations, AMLR vs. AMLR Table S7 - S8**

**Correlations, ABR vs. OAE Table S9**

**Correlations, AMLR vs. OAE Table S10 - S11**

**Correlations, ABRs vs. Psychoacoustical Tasks Table S12 - S13**

**Correlations, AMLRs vs. Psychoacoustical Tasks Table S14 - S17**

**Correlations. Test vs. Retest Table S18**

**Additional Summary Figures *https://repositories.lib.utexas.edu/handle/2152/69854 or http://doi.org/10.15781/T29G5H02*R**

**ABRs Fig. S1 - S7**

**AMLR, Latency Fig. S8 - S14**

**AMLR, Amplitude Fig. S15 - S19**

**Add'l Averaged-Waveform Figures *https://repositories.lib.utexas.edu/handle/2152/81116***

***or http://dx.doi.org/10.26153/tsw/8129***

**Template for scoring Fig. S20**

**Sex differences Fig. S21 - S33**

**Race differences Fig. S34 - S39**

**Menstrual-cycle differences Fig. S40 - S45**

**Effect of click level Fig. S46**

**Lack of latency/amplitude shift Fig. S47 - S48**

**Raw Data for AEPs, OAEs, Behavior *http://hdl.handle.net/2152/72393***

***or http://doi.org/10.15781/T2N58D668***

DISCUSSION IVsupp

Sex differences IVsupp.A

Summary IVsupp.A.1

**Specifics IVsupp.A.2**

Race differences IVsupp.B

Summary IVsupp.B.1

Specifics IVsupp.B.2

Two comments IVaupp.B.3

Menstrual-cycle differences IVsupp.C

Summary IVsupp.C.1

**Specifics IVsupp.C.2**

**Lack of level effect IVsupp.D**

Correlations between AEPs, and between AEPs and OAEs IVsupp.E

Correlations between AEPs and psychoacoustical performance IVsupp.F

Greater male variability IVsupp.G

Final comments IVsupp.H

**REFERENCES**

***Supplementary text in BOLD**

Supplement to

Auditory evoked potentials: Differences by sex, race, and menstrual cycle and correlations with common psychoacoustical tasks

Dennis McFadden^a,b^, Craig A. Champlin^c^, Michelle H. Pho^c^, Edward G. Pasanen^b^,

Mindy M. Maloney^b^, Erin M. Leshikar^c^

^b^ *Department of Psychology and Center for Perceptual Systems, University of Texas, 108 East Dean Keeton, A8000, Austin, Texas 78712-1043*

^c^ *Department of Speech, Language, and Hearing Sciences, University* *of Texas,*

*2504-A Whitis Avenue, A1100, Austin, Texas 78712-0114*

^a)^ Author to whom correspondence should be addressed. Electronic mail: mcfadden@utexas.edu

**Submitted to *Public Library of Science (PLOS ONE)*, 10/7/20**

## Do Not Cite

**ABSTRACT**

**see published article**

**Isupp. INTRODUCTION**

**see published article**

**Isupp. A. Context**

**see published article**

*PLOS formatting forbids the use of page numbers in citations. In this section, the various points about AEPs attributed to Hall (2015), citation [3], can be found (in order mentioned) on pages 377 (sex differences, puberty effects), 378 (head size), 202 (hearing sensitivity), 474 (race), 568 (brain regions), 606 (Waves V and Po), 491 (V/I ratio).*

**IIsupp. METHODS**

**see published article**

**IIsupp. A. Subjects**

**see published article**

Our granting agency required that these two items be asked of all subjects:

Item 1. (Yes or No) “Are you of Hispanic, Latino, or Spanish Origin (Cuban,

Mexican, Puerto Rican, South or Central American, or other Spanish culture

or origin, regardless of race)?”

Item 2. (Pick One) “Are you American Indian or Alaskan native; Asian; Black or African American; Native Hawaiian or Other Pacific Islander; White; More than one race; Other or Unknown (please specify if known)."

All subjects answering “White” on item 2 were categorized as such, and all subjects choosing any other category on item 2 were categorized as Non-White, no matter what their response to item 1.

Some of our questionnaire items provided information about sexual orientation. No one was excluded from the study because of sexual orientation, but the data from 7 female and 5 male non-heterosexual subjects were excluded from all the analyses reported here because of past findings (McFadden and Champlin, 2000) and to be consistent with our two previous reports (McFadden et al., 2018a, b). This decision means that here "sex" actually *is* synonymous with "gender," under the scientific definitions of those terms. The sexual orientations of the subjects were not known to the daily experimenters. A separate experimenter was responsible for recording the questionnaire responses and often those responses were not recorded until data collection for that crew was complete.

**see published article**

**IIsupp. B. General procedures**

**see published article**

***IIsupp. B. 1. Menstrual cycle***

Female crews worked for 8 - 10 weeks, during which they kept daily diaries about their menstrual cycles. After all the data were collected, the diary entries were used to partition each individual subject’s daily psychoacoustical data into three categories: Menses, Midluteal, and the remainder (nominally Ovulatory). All definitions were tied to the first day of menses as follows: All blocks of trials collected during the first 5 days of a cycle were defined as Menses, all blocks collected between 3 and 12 days prior to the onset of menses were defined as Midluteal, and all blocks collected between day 6 and the 12th day before the next menses were defined as Ovulatory; for additional detail see McFadden et al. (2018a). The onset of menses was defined as the first day of reported bleeding, which often occurred one day after the first report of spotting.

Collecting the physiological data for the females was more complicated than for the males. In addition to keeping daily diaries, female subjects also kept the (all-female) experimenters orally informed about each onset of menses. When a subject’s period began, one measurement session for OAEs, ABRs, and AMLRs was immediately scheduled, as was a second session at a time predicted to fall within the definition of that subject's midluteal phase. The subject’s self-reports of previous cycles were used to predict the time of the second (midluteal) session. If the onset of the next menses revealed that the prediction about the midluteal phase had been in error, that session was discarded and another was collected during the following cycle.  When an agreeable time could not be found outside the daily 2-hr window during which the psychoacoustical data were collected, then OAEs, ABRs, and AMLRs were measured during the crew time (while the rest of the crew was being tested on psychoacoustics).

**see published article**

**IIsupp. C. Measuring auditory evoked potentials**

**see published article**

**IIsupp. D. Measurement details for ABRs and AMLRs**

**see published article**

Prior to the final analyses, 0.9 ms was subtracted from the mean latencies of all subjects to remove both the delay introduced by the acoustic path between earphone and cochlea and the delay in the bioamplifiers (McFadden, Garcia-Sierra, et al., 2010, 2012a).

To aid the judge in scoring waveforms, LabVIEW^®^ software (National Instruments, Austin, TX) was developed by author EGP. that displayed one of four template waveforms along with the waveform to be scored. The template that appeared was either ABR or AMLR for either 70-dB click or 40-dB click. Each template was an average of the individual waveforms recorded from 10 adults (5 females, 5 males), each of whom clearly exhibited all components of the ABR and AMLR responses. Those 10 exemplary responses were obtained under conditions identical to those used in the present study, but from subjects who did not participate in this study. The template waveforms were used only as aids to scoring; the judge still was responsible for identifying peaks by eye. Following identification, the software sought the local inflection point in the wave and used that point to calculate latency and amplitude. Two of the ABR and AMLR templates are shown as Figure **S20** here.

To assess the reliability of the judge, a pseudorandom sample of waveforms was re-scored. In all, the latencies for 570 peaks pooled across ABR, AMLR, two click levels, sex, and race were blindly re-scored, typically with weeks between the two scorings. When all those pairs of scores were pooled for one calculation, the test/retest correlation was 0.99, and for 92% of those pairs, the test and retest scores were *identical*, demonstrating that reliability of scoring was excellent. When the test/retest correlations instead were calculated for single peaks, they were higher for Wave V than for Wave I (0.96 vs. 0.43), the same for Wave V and the peaks of the AMLR (~0.91), higher for the 70-dB click than for the 40-dB click (0.96 vs. 0.85), and the same for the two sexes (see Table **S18** here). All scoring and rescoring was done blind as to race, phase of the menstrual cycle, and the first scoring.

Another reflection of the high reliability of the judge is that the lack of significant differences in the AEP data across the menstrual cycle was *not* attributable to high variability, as the error bars show. On the contrary, the AEPs *and their scoring* were nearly identical for both phases of the cycle.

**IIsupp. E. Two complications**

After several all-male crews had been tested, the true rms voltmeter (VM) used for setting the levels of the stimuli for the psychoacoustical tasks malfunctioned (VMm). As a consequence, those early male crews were tested with stronger stimuli than used for the remainder of the male crews and for all of the female crews. Thus, the *psychoacoustical* data obtained from those early subjects could not be pooled with the psychoacoustical data from the later crews, neither for the analyses reported in McFadden et al. (2018a, b), nor for those comparisons reported here that involve the psychoacoustical tasks. Accordingly, when analyzing the relationships between AEPs and psychoacoustical tasks, we use only the data from the Post-VMm males for comparisons with the females. Because the malfunctioning VM was not used in the measurement of AEP (or OAE) stimuli, when discussing only the AEP (or OAE) measures, we use the male data pooled over the Pre-VMm and Post-VMm groups (typically labeled “all males”). For completeness and consistency with McFadden et al. (2018a, b), all the figures show the male data both partitioned by VMm and pooled over VMm, and the Ns are shown for all the groups. (Again, all female crews were tested after the malfunction of the VM.)

**see published article**

**IIsupp. F. Statistical analyses and resampling**

**see published article**

Because the number of correlations calculated was large, it was necessary to assess which of them were the most *un*likely to be due to chance. Again, we used a resampling technique. To be specific, correlations were calculated for all of the pairwise comparisons of interest; we call these the *actually obtained correlations*. Then, for each pair of conditions being compared, the obtained values for the first variable were retained for each subject, but all the values for the second measure were replaced at random by values achieved by *other* subjects in that same group. That correlation was calculated and saved (called a resampled correlation), the process of randomly reassigning the second value was repeated 20,000 times, and a tally was kept of how often the resampled correlation equaled or exceeded the actually obtained correlation for that comparison. That tally was divided by 20,000 to yield a proportion called the *implied significance* for the actually obtained correlation, an estimate of how rare the actual outcome was. As with effect sizes, we used the absolute value of the resampled correlations for our tallies, so again our estimates of implied significance are conservative ("two-tailed"). Cohen (1992) suggested that correlations of 0.1, 0.3, and 0.5 be viewed as small, medium, and large effects, respectively.

**IIsupp. G. Averaged waveforms**

***IIsupp. G. 1. Description***

**see published article**

***IIsupp G. 2. Weaknesses of averaged waveforms***

We believe that averaged waveforms have several weaknesses as a way of summarizing AEP data, and we regard the peak-by-peak analyses presented in the published figures and tables to be superior to averaged waveforms.

One weakness of averaged waveforms is that they typically contain no information about the variability of the individual peaks, and if "bands" are added to the average traces to indicate variability, it becomes difficult to present two or more averaged waveforms together for comparison. Clearly, information about variability is crucial for determining the significance of any differences visible in the averaged waveforms.

Second, with our peak-by-peak analyses, when an expected peak is absent or anomalous in an individual waveform, the measurements for that single peak for that individual waveform can be excluded from the analysis while the measurements for all the other (typical-appearing) peaks for that individual are included. When averaging waveforms, there is no mechanism for selective exclusions of this sort; waveforms are either included or excluded in their entirety. This means that it is possible for the shapes of two averaged waveforms to differ only because of differences in the number of subjects having anomalous peaks contributing to the two averages. The greater flexibility of the peak-by-peak procedure allows the use of as much of the collected data as possible.

One other weakness of averaged waveforms is that the shape of the average can differ from the shape of the individual waveforms from which it is composed. For example, all the individual ABR waveforms contributing to the four averaged waveforms in Fig. 4 (McFadden et al., 2020) were examined for the presence of a Wave IV that was visibly distinct from Wave V. The result was that Wave IV was present in 78% of the subjects, yet none of the four averaged waveforms in Fig. 4 exhibits a clear Wave IV. When characteristics possessed by the majority of the individual subjects can be lost in the averaging process, such averaging becomes problematic as an appropriate summary of the results. (The loss of Wave IV when ABR waveforms are averaged across subjects may provide a partial explanation for why, historically, this peak has not been commonly studied.)

Although we believe peak-by-peak analyses are preferable to averaged waveforms for summarizing a set of AEP results, averaged waveforms can provide helpful illustrations of certain outcomes or group differences. Accordingly, we have provided several figures with averaged waveforms, each comparing a different pair of subject groups or conditions (see Figs. 4 and 5 in the published article and Figs. **S21** - **S45** here).

In accord with the strategy adopted here, Gallun et al. (2012) also reported means and SDs for latency and amplitude for each peak of interest and presented averaged waveforms only as illustrative. If an individual amplitude could not be determined for a particular subject, only that amplitude was omitted for that subject, as was done here (also see Krizman et al., 2019).

**IIsupp.H. Strategy**

**see published article**

**IIIsupp. RESULTS**

**see published article**

**IIIsupp. A. ABRs: Sex, race, and menstrual-cycle differences**

***IIIsupp. A. 1. Overview***

**see published article**

***IIIsupp. A. 2. Representative ABR outcome***

**see published article**

***IIIsupp. A.3. Remainder of ABR measures***

**see published article**

**IIIsupp. B. AMLRs: Sex, race, and menstrual-cycle differences**

***IIIsupp. B. 1. Overview***

**see published article**

***IIIsupp. B. 2. AMLR latency***

**see published article**

***IIIsupp. B. 3. AMLR amplitude***

**see published article**

Discussion of Fig. 3: When the subjects were pooled over race (top panels), the mean amplitude for females was significantly smaller than for the males (effect size = -0.72; *p* = 0.0006). When the subjects were partitioned by race (bottom panels), the sex differences persisted, but the mean amplitudes for males and females were visually more different for the Non-White subjects (effect size = -0.66; *p* = 0.02) than for the White subjects (effect size = -0.53; *p* = 0.14). Evident in Fig. **3** is a pattern also seen in Fig. **2**; the mean amplitudes ordered differently for the Post-VMm and Pre-VMm males in the two race groups. This pattern existed for 5 of the 6 AMLR amplitude measures. Unfortunately, when the subjects are partitioned into pre-VMm and post-VMm groups, the Ns become rather small. Figures for each of the other AMLR amplitude measures can be found here (Figs. **S15** – **S19**).

Discussion of Table 3: As previously noted, only a few comparisons between the sexes achieved implied significance, but all the effect sizes were numerically larger for the Non-White subjects. In accord with a trend seen for the AMLR-latency measures (Table 2), the effect sizes for sex difference tended to be larger with the weaker click than with the stronger click for the later waves [column (3) *vs*. column (4), and column (5) *vs*. (6)]. As with all the other AEP measures shown so far, no comparison for race or menstrual cycle achieved implied significance.

**IIIsupp. C. Averaged waveforms**

**see published article**

**IIIsupp. D. Correlations between AEP measures**

***IIIsupp. D. 1. ABRs***

**see published article**

Although overall patterns are difficult to discern in Table 4, some pairs of conditions exhibited generally similar, and at least moderately high, correlations for both sexes and race groups. The primary examples are Wave-V latency, 70 dB *vs*. 40 dB; Wave-I amplitude *vs*. the amplitude ratio, Wave V/Wave I; Wave-V amplitude *vs*. the amplitude ratio, Wave V/Wave I; and Wave-V latency, 70 dB *vs*. the interpeak interval, Wave I -> Wave V. Admittedly, the final three examples are not especially surprising because in each case the first measure contributed to the second, composite measure. Other pairs of conditions showing similarity across the sexes are: Wave-V latency *vs*. Wave-V amplitude (for both click levels); and Wave-V amplitude, 70 dB *vs*. Wave-V amplitude, 40 dB. Other pairings expected to be highly correlated typically were, but often not for both sexes or both races.

***IIIsupp. D. 2. AMLRs***

**see published article**

Intercorrelations like those in Table 4 also were tabled for the 8 AMLR **latency** measures, using the same format; see Table **S1**. In summary, there were fewer high correlations than for ABRs (see Table 4). In Table **S1**, the correlations generally were larger for the females than for the males, and the tendency was for slightly higher correlations for the White subjects than for the Non-White subjects. Three pairs of conditions showed high, significant correlations for both sexes and both race groups; they were: Wave-Po latency, 70 dB *vs*. 40 dB; Wave-Na latency, 70 dB *vs*. 40 dB; and Wave-Nb latency, 70 dB *vs*. 40 dB. The remainder of the pairs of conditions having correlations that achieved implied significance did so primarily for one sex or race group.

Intercorrelations like those in Table 4 also were tabled for the 6 AMLR **amplitude** measures; see Table **S2**. To summarize the results, more correlations achieved implied significance for the males than for the females, and the correlations generally were larger for the Non-White subjects than for the White subjects. Five pairs of conditions showed high, significant correlations for both sexes and both race groups; they were: Wave Po-Na, 70 dB *vs*. 40 dB; Wave Na-Pa, 70 dB *vs*. 40 dB; Wave Pa-Nb, 70 dB *vs*. 40 dB; Wave Po-Na, 70 dB *vs*. 70 dB; and Wave Na-Pa, 40 dB *vs*. Wave Pa-Nb, 40 dB. The males had several other pairs of conditions that achieved implied significance for one or both race groups, but the corresponding correlations for the females were non-significant.

***IIIsupp. D. 3. Other correlations***

Also calculated were the correlations between the various ABR measures and the various AMLR measures. Tables **S3** and **S4** show the correlations between the 8 ABR measures and the 8 AMLR latency measures, one table for each sex. Note that the latencies for Waves V and Po were well-correlated for both sexes, as were the amplitudes of those two waves. Tables **S5** and **S6** show the correlations between the 8 ABR measures and the 6 AMLR amplitude measures, and Tables **S7** and **S8** show the correlations between the 8 AMLR latency measures and the 6 AMLR amplitude measures. The patterns of results were not consistent across sex or race.

Also calculated were the correlations between the various AEP measures and the various OAE measures. OAEs are products of the cochlea and AEPs are products of gross neural activity. Logic and intuition suggest that the former might affect the latter, particularly for the ABRs. Table **S9** shows the correlations between the 8 ABR measures and the 4 OAE measures (SOAEs, CEOAEs collected with two click levels 6 dB apart, and DPOAEs); Table **S10** shows the correlations between the 8 AMLR latency measures and the 4 OAE measures; Table **S11** shows the correlations between the 6 AMLR amplitude measures and the 4 OAE measures. These correlations were weak for both sexes both within and across race groups, and, contrary to intuition, the correlations between OAEs and the waves of the ABR were not stronger than those between OAEs and the waves of the AMLR.

When evaluating these correlations, the reader needs to appreciate that spontaneous OAEs (SOAEs) have a weakness when it comes to correlational analyses; namely, many ears have zero SOAEs, yielding a truncated distribution. CEOAEs and DPOAEs are free of this weakness.

One of the primary initial objectives for this large-scale study was to determine whether performance on any of a set of common psychoacoustical tasks was correlated with any of our OAE measures. The results were underwhelming (McFadden et al., 2012b; McFadden et al., 2018a, b). A second initial goal was to determine whether psychoacoustical performance would correlate with any of our AEP measures.

A list of the seven psychoacoustical tasks measured was given in McFadden et al. (2018a). As discussed there, the psychoacoustical tasks covered a range of masking conditions, both simultaneous and temporal, as well as one informational-masking task (profile analysis), all in the 3.0-kHz region. The tasks were chosen because previous research had linked them to the active process in the cochlea (cochlear amplification) or had shown them to exhibit a large sex difference. The potential for strong correlations with OAEs seemed high; the possibility of strong correlations with AEPs was far less clear.

Because of the malfunction of the VM, the comparisons between AEPs and psychoacoustical performance necessarily are restricted to the Post-VMm males, a smaller N than used for all the correlations presented to this point. The unanticipated necessity to partition the subjects by race further diminished the Ns. Because of the notable absence of any differences in AEPs or behavior across the phases of the menstrual cycle, the female data used for correlations were those averaged across the cycle. The correlations are presented with the subjects both pooled across race and partitioned by race.

The outcome was that no correlations between individual AEP measures and individual psychoacoustical tasks were large and consistent across the race groups or sexes. The few correlations that did achieve implied significance appeared to be chance occurrences because of the lack of consistency across sex and race groups. For whatever reasons, more correlations again achieved implied significance for the White subjects than for the Non-White subjects. The appropriate generalization appears to be that our AEP measures were not related to our psychoacoustical measures. The correlations are presented in Tables **S12** - **S17**.

As noted, when an occasional correlation between an AEP measure and a psychoacoustical task did achieve implied significance, typically it did so for only one sex or only for one race group (presumably by chance). Two exceptions occurred: the correlation between Wave-Na latency at 70 dB and the width of the auditory filter achieved implied significance for the White subjects of both sexes, and the same was true for Wave-Pa latency at 70 dB *vs*. the width of the auditory filter. Neither past research nor intuition provide any guidance as to whether these relationships might replicate.

**IVsupp. DISCUSSION**

*PLOS formatting forbids the use of page numbers in citations. In this section, the various points about AEPs attributed to Hall (2015), citation [3], can be found (in order mentioned) on pages 377 (sex differences), 270+ (stimulus magnitude), 339 (stimulus magnitude), 579 (stimulus magnitude), 222 (auditory ability), 446 (hearing sensitivity), 232 (frequency).*

**IVsupp. A. Sex differences**

***IVsupp. A. 1. Summary***

**see published article**

***IVsupp. A. 2. Specifics***

**see published article**

The large sex difference for the interpeak interval Wave I -> Wave V (also see Jerger and Hall, 1980; Chan et al., 1988; Hall, 2015, p. 377), may simply be an arithmetic consequence of the large sex difference in Wave-V latency.

**IVsupp. B. Race differences**

***IVsupp. B. 1. Summary***

**see published article**

***IVsupp. B. 2. Specifics***

**see published article**

***IVsupp. B. 3. Two comments***

**see published article**

We have some additional comments on the role of race in the sex-by-race interactions reported here and in McFadden et al. (2018a, 2018b). Our strong belief is that, in time, race will prove to be irrelevant to our results. It is only a temporary, proxy measure for the factors or mechanisms actually producing the race effects reported. The argument follows:

Modern medicine and science are moving slowly toward studying and treating individual differences -- so-called personalized or precision medicine. The goal is to identify the genetic, metabolic, or whatever factors or mechanisms that underlie various medical conditions (or scientific findings). Then, once identified, those factors or mechanisms will be measured directly, and a diagnosis and path of treatment chosen accordingly. Currently, those specific factors or mechanisms are not fully determined for most medical conditions, so instead, diagnosis and treatment (or scientific interpretation) must rely on proxy measures. An imaginary example might help: People with *green* hair are more likely to have Malady Z than are other groups of people, so when a person with green hair presents with symptoms X and Y, the assumption is that Malady Z is the reason, and treatments appropriate for Malady Z are prescribed. If a person with *purple* hair presents with symptoms X and Y, a different diagnosis and treatment might be reached -- because Malady Z is known to be far less common in people with purple hair than in people with green hair. Thus, hair color serves as a temporary proxy for the actual underlying genetic, metabolic, or whatever factors or mechanisms are responsible for Malady Z. Once the underlying factor or mechanism *are* known, they can be measured directly to determine the appropriate diagnosis and treatment, and then there will be no need to consider hair color.

In the analogy then, here race is only a temporary proxy in the various sex-by-race Interactions in hearing measures. There are some factors or mechanisms that are responsible for, say, strength of the cochlear amplifiers, and those genetic or metabolic factors or mechanisms just happen to be more common in Non-White males and females, and that leads to the smaller sex differences we observed in our Non-White group. The factors or mechanisms *are* present in both Non-Whites and Whites; they are just more common in Non-Whites -- hence the group differences we have reported. Eventually, however, the true cause of individual differences in the strength of the cochlear amplifiers will be known, and scientists will measure that factor directly when trying to explain some scientific outcome. We will not need to appeal to the temporary proxy of race. Race will become irrelevant.

The importance of acknowledging race differences at this stage of our ignorance is that the race differences can be a clue to the nature of those elusive factors or mechanisms actually underlying the differences observed. In our opinion, not reporting the sex-by-race interactions we observed would have been scientifically dishonest. But, our belief is that, eventually, race will prove to be irrelevant to the explanation of results like ours. Race is simply correlated with the relevant underlying factors or mechanisms.

Recall that in this study, the two race categories were necessarily so ill-defined that Asians of various origins, Native Americans, Pacific Islanders, and Blacks all were pooled into the Non-White group. Clearly, whatever the relevant factor or mechanism producing the group differences we observed proves to be, it will not be "race."

**IVsupp. C. Menstrual-cycle differences**

***IVsupp. C. 1. Summary***

**see published article**

***IVsupp. C. 2. Specifics***

**see published article**

Considerable effort was expended in this study on the topic of the menstrual cycle.  Some primary initial goals of the study were to confirm past reports about differences across the cycle (e.g., Swanson and Dengerick, 1988; Elkind-Hirsch et al., 1992a, b; Upadhayay et al., 2014), to determine if they extended to additional physiological and behavioral measures, and to document the magnitude of those differences.  The first author previously had reviewed the existing literature on auditory changes across the menstrual cycle (McFadden, 1998) and had tried to organize the various reports into a consistent story.  One of the weaknesses of past studies was an inconsistent definition of the phases of the cycle. The evidence reviewed by McFadden (1998) made it appear that stronger menstrual differences were associated with the midluteal phase than the ovulatory phase. Accordingly, the menses and midluteal phases were studied for this work. (The ovulatory phase is short and difficult to identify with precision; also, a substantial number of young women do not ovulate on every cycle.) Estrogen levels are high immediately preceding ovulation and then drop and rise again so that both estrogen and progesterone are high in the midluteal phase.

As explained in Section II.B.1, all our female subjects were naturally cycling and each kept a detailed diary of her cycles during this study. The diary entries were used *after* all data were collected to partition the daily psychoacoustical data into Menses and Midluteal phases. Also, the onset of menses was used to determine when the individual AEP/OAE test sessions were scheduled.

In the end, this considerable effort resulted in no compelling evidence of an effect of the menstrual cycle on any of our physiological or psychoacoustical measures (see McFadden et al., 2018a, b, 2020).  Special surprise was associated with the lack of convincing cycle differences for Wave V of the ABR because previous results seemingly were definitive about different latencies at different points in the cycle (Elkind-Hirsch et al., 1992a, b; Upadhayay et al., 2014). Similar results for event-related potentials (Tillman, 2010) strengthened our expectations that AEPs would differ across the cycle, yet no cycle differences were found for Wave-V latency or any other of our AEP measures. Circumstantial evidence that cyclic hormonal fluctuations ought to affect AEPs and OAEs is that use of oral contraceptives affects both those physiological measures (McFadden, 2000).

One possible explanation for this failure to replicate is that Elkind-Hirsch et al. (1992a, b) and Tillman (2010) compared measures made during menses with measures made during the *ovulatory* phase, not midluteal as here. (Upadhayay et al., 2014, compared pre-ovulatory and mid-luteal measures.) Our emphasis on the midluteal phase may have been a mistake, originating with the McFadden (1998) review. We might have measured larger differences across the cycle here, at least for ABRs, had we compared the menses phase with the ovulatory phase rather than with the midluteal phase.

Other possible explanations for our general failure to replicate and extend cycle differences may lie in our procedures. All of our psychoacoustical tasks tested the 3.0-kHz region, and many previous studies used lower frequency signals. Our subjects were highly practiced on all the psychoacoustical tasks, something generally not true of the subjects in past behavioral studies of the menstrual cycle.  With practice on psychoacoustical tasks comes a degree of automaticity, and that may have served to make our subjects more consistent in their psychoacoustical performance across the cycle than were subjects in past studies. Similarly, our AEP (and OAE) measures were made multiple times on each subject, so any novelty effects that might have affected our physiological measures surely were reduced. Our female subjects surely were aware that we anticipated differences across the menstrual cycle (why else were they keeping personal diaries about their cycle?). However, they did not know what direction of effect was anticipated, and even if they guessed correctly, we know of nothing they could have done to alter their AEP (or OAE) measures even if they did have a theory about our anticipations. What is unsatisfactory about appeals to practice and novelty as explanations for past findings is the necessity for the *lack* of practice and/or novelty in past studies to have operated differentially on measurements made during different phases of the cycle.

Whatever the ultimate explanation for our failure to observe marked effects of the menstrual cycle, the open-science initiative (Open Science Collaboration, 2015; Nosek et al., 2015) requires that our null effects be reported.

***IVsupp. D. Lack of level effect***

**see published article**

One of the most commonly reported effects in the study of sensory systems is that increases in stimulus strength lead to both a decrease in the latency between the stimulus and response and an increase in the amplitude of that response. This effect has been reported for many physiological (e.g., Dallos, 1985; Cody and Russell, 1987; Recio et al., 1998; Baudin et al., 2019) and behavioral (e.g., Pins and Bonnet, 1996) measures. The effect typically diminishes at high stimulus levels. Here we denote these twin effects as the latency/amplitude shift; depending upon the context, other terms are Piéron's Law, latency-intensity function, and others. For simple auditory stimuli, one contributing factor to the latency change would be the basalward migration of the traveling-wave envelope with increasing stimulus strength, a decrease in propagation time (McFadden, 1986).

Latency/amplitude shifts were observed early in the study of AEPs (e.g., Hecox and Galambos, 1974; Hall, 2015, p. 270+, p. 339, p. 579). They generally were evident in the present data, but not for the measures involving Wave Pa. Because exceptions to the typical finding are so rare, and because of the open-science initiative, a discussion of those results is in order.

Although averaged waveforms have serious weaknesses (see section IIsupp G. 2), the waveforms seen in Figs. **S31** and **S46** do help illustrate the typical and anomalous outcomes of click level. For both figures, the females are pooled across both race and the menstrual cycle, and males are pooled across race; the waveforms for the 70-dB and 40-dB clicks are in the top and bottom panels, respectively. Within each figure, a comparison of the ordinate values on the two panels reveals that the responses generally were weaker to the 40-dB click than to the 70-dB click, and a comparison of the abscissa values reveals that the responses to the 40-dB click generally were slid toward longer latencies than for the 70-dB click. So, in general latency/amplitude shifts did exist in these data. (However, in Fig. **S46**, note the similarities in peak amplitude and latency across level beginning about 20 ms post-click.)

Quantitative summaries are contained in Figs. **S47** and **S48**. Fig. **S47** shows differences in *amplitude* across the two click levels for several AEP peaks. Attend first to the All-subjects data at the far left of the figure. For both sexes, the difference in amplitude for Wave Pa-Nb was quite small compared to those for the other peaks, and this pattern persisted when the subjects were partitioned by race (middle and right sections of the figure).

Fig. **S48** shows differences in *latency* across the two click levels for those same AEP peaks. Attend first to the All-subjects data at the far left of the figure. The latency difference for Wave Pa is smaller than for the other peaks shown, but the effect is more marked for females than for males. That pattern holds when the data are partitioned by race (middle and right sections of the figure).

That is, the commonly seen differences in amplitude and latency when click level is varied were essentially absent for both sexes for the amplitude of Wave Pa-Nb and absent for females for the latency of Wave Pa. That is, sex mattered for latency, but not for amplitude.

Unlike here, Tucker and Ruth (1996) did find the canonical increase in amplitude and decrease in latency for Wave Pa for both teenagers and adults using two click levels similar to those used here. The stimulus-presentation rates were different from those used here, and the data were not partitioned by sex or race, but the canonical effects existed over a wide range of ages. Hall (2015, p. 579) discussed several studies in which AMLR responses behaved in accord with the prototypical responses to level changes only over a restricted range of level. Perhaps our findings are attributable to a complex interaction between click levels and individual subjects. Again, our click levels were about 75 and 105 peak-equivalent dB SPL, corresponding to about 40 and 70 dB nHL, and all measurements were for the right side of the head only.

Because ABRs and AMLRs are sums of synchronous neural activation across large segments of the brain (Møller et al., 1995), it is not difficult to generate plausible speculations about the reason for the absence of amplitude or latency changes across click levels, especially for a wave that occurs so long after the stimulus. For example, Wave Pa is strong and quite broad (see Fig. **S20**). It is logically possible that the breadth of the wave is the result of multiple sub-populations of neurons (possibly from different locations in the brain) firing in close succession (and possibly firing multiple times each). As stimulus level is increased, imagine that the latencies of some sub-populations of neurons do decrease but their firing rates also saturate, meaning that those sub-populations now contribute less to the overall peak of Wave Pa. Also imagine that other sub-populations of neurons, which have a longer natural latency to the stimulus, do not experience saturation until much higher stimulus levels. The contribution of the latter sub-populations to Wave Pa would increase with increasing stimulus level, with the result that latency and peak amplitude would be relatively unchanged for a particular pair of stimulus levels. Factors such as these may be responsible for AMLR peaks being more highly dependent upon the details of the stimulus than are ABR peaks (Tucker et al., 2002). Note that speculations of this sort are not necessary for any of the early waves of the AMLR because they do show evidence of changes with click level.

The absence of substantial sex differences for latency for Waves Na and Pa is interesting given that the preceding wave (Po) and the subsequent wave (Nb) both did exhibit large sex differences. This pattern of results reveals that the neural locations giving rise to this sequence of waves are not simply wired in series (also see McFadden and Champlin, 2000; McFadden et al., 2010). If they were strictly wired in series, one would expect that any early advantage in latency acquired by the females would be passed to successive neural locations essentially unchanged; the sex differences in latency would remain similar across successive links in the auditory chain. Rather, it appears that different neural channels of information are operating in parallel and contributing differentially to the latencies and amplitudes of successive waves. That is, it appears as if some neural channel carries timing information from the location(s) responsible for Wave Po to those responsible for Wave Nb without contributing much if anything to those locations responsible for Waves Na and Pa. (The alternative is that sex differences in latency arise *de novo* at successive locations in the auditory chain, rather an unlikely prospect.) Also relevant to the issue of successive neural locations are the findings and discussion in McFadden et al. (2010). In this context, note that only one of the ALR waves that follow the AMLR waves exhibits a sex difference for latency (McFadden and Champlin, 2000).

In summary, both the latency and amplitude of some of the latest waves measured here did not exhibit the effects of level so commonly observed for physiological measures of this sort, and the discrepancy appeared to be more marked for amplitude than latency, and more marked in females than males.

**IVsupp. E. Correlations between AEPs, and between AEPs and OAEs**

**see published article**

**IVsupp. F. Correlations between AEPs and psychoacoustical performance**

**see published article**

**IVsupp. G. Greater male variability**

**see published article**

**IVsupp. H. Final comments**

**see published article**

Through the lens of hindsight, essentially every failed experiment can be seen as fatally flawed from its outset.

**ACKNOWLEDGMENTS**

**see published article**

**REFERENCES**

**see published article**

Baudin, J., Angueyra, J. M., Sinha, R., and Rieke, F. (**2019**). "S-cone photoreceptors in the primate retina are functionally distinct from L and M cones," eLife, **8**, e39166.

Chan, Y. W., Woo, E. K. W., Hammond, S. R., Yiannikas, C., and McLeod, J. G. (**1988**). “The interaction between sex and click polarity in brain-stem auditory potentials evoked from control subjects of Oriental and Caucasian origin,” Electroencephalog. Clin. Neurophysiol. **71**, 77-80.

Cody, A. R., and Russell, I. J. (**1987**). "The responses of hair cells in the basal turn of the guinea-pig cochlea to tones," J. Physiol. **383**, 551-569.

Cohen, J. (**1992**). “A power primer,” Psychol. Bull. **112**, 155-159.

Dallos, P. (**1985**). "Response characteristics of mammalian cochlear hair cells," J. Neurosci. **5**, 1591-1608.

Elkind-Hirsch, K. E., Stoner, W. R., Stach, B. A., and Jerger, J. F. (**1992a**). “Estrogen influences auditory brainstem responses during the normal menstrual cycle,” Hear. Res. **60**, 143-148.

Elkind-Hirsch, K. E., Wallace, E., Stach, B. A., and Jerger, J. F. (**1992b**). “Cyclic steroid replacement alters auditory brainstem responses in young women with premature ovarian failure,” Hear. Res. **64**, 93-98.

Gallun, F. J., Diedesch, A. C., Kubli, L. R., Walden, T. C., Folmer, R. L., Lewis, M. S., McDermott, D. J., Fausti, S. A., Leek, M. R. (**2012**). "Performance on tests of central auditory processing by individuals exposed to high-intensity blasts," J. Rehabil. Res. Dev. **49**, 1005–1024.

Hall, J. W., III. (**2015**). *eHandbook of auditory evoked responses: Principles,*

*procedures and protocols.* New York: Kindle Direct Publishing, pp. 1122. ASIN: B011J59898

Hecox, K., and Galambos, R. (**1974**). "Brain stem auditory evoked responses in human infants and adults," Arch. Otolaryngol. **99**, 30-33.

Jerger, J., and Hall, J. (**1980**). “Effects of age and sex on auditory brainstem response,”

Arch. Otolaryngol. **106**, 387-391.

Krizman, J., Bonacina, S., Kraus, N. (**2019**). "Sex differences in subcortical auditory processing emerge across development," Hear. Res. **380**, 166-174.

McFadden, D. (**1986**). "The curious half-octave shift: Evidence of a basalward migration of the traveling-wave envelope with increasing intensity." In R. Salvi, D. Henderson, R.P. Hamernik and V. Coletti (eds.), *Applied and Basic Aspects of Noise-Induced Hearing Loss*. New York: Plenum, p, 295-312.

McFadden, D. (**1998**). “Sex differences in the auditory system,” Dev. Neuropsychol. **14**,

261-298.

McFadden, D. (**2000**). "Masculinizing effects on otoacoustic emissions and auditory evoked potentials in women using oral contraceptives," Hear. Res. **142**, 23-33.

McFadden, D., and Champlin, C. A. (**2000**). “Comparison of auditory evoked potentials in heterosexual, homosexual, and bisexual males and females,” J. Assoc. Res. Otolaryngology **1**, 89-99.

McFadden, D., Champlin, C. A., Pho, M. H., Pasanen, E. G., Maloney, M. M., Leshikar, E. M. (**2020****). "Auditory evoked potentials: Differences by sex, race, and menstrual cycle and correlations with common psychoacoustical tasks,"

PLOS, *******, ***-***.

McFadden, D., Hsieh, M. D., Garcia-Sierra, A., and Champlin, C. A. (**2010**).

“Differences by sex, ear, and sexual orientation in the time intervals between

successive peaks in auditory evoked potentials,” Hear. Res. **270**, 56-64.

McFadden, D., Pasanen, E. G., Leshikar, E. M., Hsieh, M. D., and Maloney, M. M.

(**2012b**). “Comparing behavioral and physiological measures of combination tones: Sex and race differences,” J. Acoust. Soc. Am. **132**, 968-983.

McFadden, D., Pasanen, E. G., Maloney, M. M., Leshikar, E. M., and Pho, M. H.

(**2018a**). “Differences in common psychoacoustical tasks by sex, menstrual cycle, and race,” J. Acoust. Soc. Am. **143,** 2338-2354.

McFadden, D., Pasanen, E. G., Maloney, M. M., Leshikar, E. M., and Pho, M. H.

(**2018b**). “Correlations between otoacoustic emissions and performance in common psychoacoustical tasks,” J. Acoust. Soc. Am. **143,** 2355-2367.

Møller, A. R., Jho, H. D., Yokota, M., and Jannetta, P. J. (**1995**). "Contribution from

crossed and uncrossed brainstem structures to the brainstem auditory evoked

potentials: a study in humans," Laryngoscope **105**, 596-605.

Nosek, B. A., Alter, G., Banks, G. C., Borsboom, D., Bowman, S. D., Breckler, S. J., Buck, S., Chambers, C. D., Chin, G., Christensen, G., Contestabile, M., Dafoe, A., Eich, E., Freese, J., Glennerster, R., Goroff, D., Green, D. P., Hesse, B., Humphreys, M., Ishiyama, J., Karlan, D., Kraut, A., Lupia, A., Mabry, P., Madon, T., Malhotra, N., Mayo-Wilson, E., McNutt, M., Miguel, E., Levy Paluck, E., Simonsohn, U., Soderberg, C., Spellman, B. A., Turitto, J., VandenBos, G., Vazire, S., Wagenmakers, E. J., Wilson, R., and Yarkoni, T. (**2015**). "Promoting an open research culture," Science **348**, 1422-1425.

Open Science Collaboration. (**2015**). “Estimating the reproducibility of psychological science,” Science **349**, 943, aac4716. DOI:10.1126/science.aac4716.

Pins, D., and Bonnet, C. (**1996**). "On the relation between stimulus intensity and processing time: Piéron's law and choice reaction time," Percept. Psychophys. **58**, 390-400.

Recio, A., Rich, N. C., Narayan, S. S., and Ruggero, M. A. (**1998**). "Basilar-membrane responses to clicks at the base of the chinchilla cochlea," J. Acoust. Soc. Am. **103**, 1972-1989.

Swanson, S. J., and Dengerink, H. A. (**1988**). “Changes in pure-tone thresholds and

temporary threshold shifts as a function of menstrual cycle and oral contraceptives,” J. Speech Hear. Res. **31**, 569-574.

Tillman, G. D. (**2010**). “Estradiol levels during the menstrual cycle differentially affect

latencies to the right and left hemispheres during dichotic listening: An ERP study,” Psychoneuroendocrinol. **35**, 249-261.

Tucker , D. A., and Ruth, R. A. (**1996**). “Effects of age, signal level, and signal rate on the auditory middle latency response,” J. Am. Acad. Audiol. **7**, 83-91.

Tucker, D. A., Dietrich, S., Harris, S., and Pelletier, S. (**2002**). “Effects of stimulus rate and gender on the auditory middle latency response,” J. Am. Acad. Audiol. **13**, 146-153.

Upadhayay, N., Paudel, B. H., Singh, P. N., Bhattarai, B. K., and Agrawal, K. (**2014**).

“Pre- and postovulatory auditory brainstem response in normal women,” Indian J. Otolaryngol. Head Neck Surg. **66**, S133-S137.

**see published article**
